# Plasma carotenoids are inversely correlated with granulocyte counts and soluble inflammatory markers in a middle-aged population: a cross-sectional study with mediation analysis

**DOI:** 10.1186/s12916-025-04266-w

**Published:** 2025-07-15

**Authors:** Jan Neelissen, Per Leanderson, Fredrik H. Nyström, Lena Jonasson, Rosanna W. S. Chung

**Affiliations:** 1 Division of Diagnostics and Specialist Medicine, Department of Health, Medicine and Caring Sciences, Linköping University, Linköping, 581 85 Sweden; 2 Occupational and Environmental Medicine Center in Linköping, Linköping, 581 85 Sweden; 3 Division of Prevention, Rehabilitation and Community, Department of Health, Medicine and Caring Sciences, Linköping University, Linköping, 581 85 Sweden

**Keywords:** Carotenoid, Leukocyte, Granulocyte, C-reactive protein, Interleukin-18, Matrix metalloproteinase-9, Population-based cohort

## Abstract

**Background:**

High intake of fruits and vegetables is generally associated with reduced levels of inflammation. In line with this, plasma levels of carotenoids have shown inverse associations with inflammatory markers, in particular C-reactive protein (CRP) and leukocyte counts. However, it remains unclear to what extent carotenoids are associated with specific leukocyte subsets or other inflammatory markers. This study systematically assessed the inter-relationships among total and individual carotenoids, circulating leukocyte subsets, and soluble inflammatory markers in a middle-aged population.

**Methods:**

A subcohort of 1078 subjects, aged 50–64, was recruited from the Swedish CArdioPulmonary bioImage Study (SCAPIS) cohort. Leukocyte subsets were determined by whole blood flow cytometry. Five major carotenoids, namely lutein, β-cryptoxanthin, lycopene, α-carotene and β-carotene, and inflammatory markers including CRP, interleukin (IL)-6 and interleukin-18, matrix metalloproteinase (MMP)-9, and myeloperoxidase (MPO), were measured in plasma. Nutrient intake was estimated by validated food frequency questionnaires.

**Results:**

Among leukocyte subsets, only granulocytes showed independent and inverse associations with all carotenoids after adjustment. CRP, IL-18, and MMP-9 exhibited similar inverse relationships with most carotenoids. Mediation analysis revealed that the associations of carotenoids with CRP and MMP-9 were mediated by granulocyte counts. Lutein and β-cryptoxanthin remained independently associated with MMP-9 after accounting for the mediation effects of granulocyte counts. No estimated nutrient intake showed comparable associations with leukocyte subsets or inflammatory markers.

**Conclusions:**

To our knowledge, this is the largest population-based study investigating relationships between plasma carotenoids, leukocyte subsets, and soluble inflammatory markers. It provides evidence that low levels of carotenoids in plasma are linked to low-grade chronic inflammation and, furthermore, that this relationship is mediated by granulocyte numbers.

**Supplementary Information:**

The online version contains supplementary material available at 10.1186/s12916-025-04266-w.

## Background

Chronic low-grade inflammation is recognized as a key contributor to the growing prevalence of age-related diseases, such as cardiovascular disease, autoimmune diseases, and cancer. Thus, as a preventive strategy, significant interest is in managing or suppressing chronic low-grade inflammation [1]. Its prevalence generally begins to increase around middle age [2, 3]. The increase in chronic low-grade inflammation may be explained by multiple factors including repeated activation of the immune system, secretion of pro-inflammatory adipokines, and production of pro-inflammatory metabolites from the gut microbiota [1].

Pharmaceutical agents may effectively suppress inflammation; however, this approach is associated with substantial adverse effects when applied to chronic low-grade inflammation. Instead, suppressing low-grade inflammation safely and affordably through diet could be a viable option [4]. A diet high in fruit and vegetable intake has been consistently linked to lower inflammation [5]. Whether this mainly reflects a healthy lifestyle or is due to direct anti-inflammatory effects by bioactive components in the diet is still a matter of dispute. Mounting evidence from experimental animal and in vitro models indicates that carotenoids, a group of phytopigments with antioxidant effects, also exert anti-inflammatory and immunomodulatory effects [6, 7]. Also, a number of epidemiological studies have reported C-reactive protein (CRP), a well-established marker of systemic inflammation, to be inversely associated with estimated intake or plasma levels of carotenoids [8, 9]. In addition, a few clinical studies have reported inverse associations between carotenoid levels and other soluble inflammatory markers such as interleukin (IL)−6, tumor necrosis factor (TNF), and matrix metalloproteinase (MMP)−9 [6, 7].

An inverse relationship between plasma carotenoids and circulating leukocyte counts has also been reported in population-based cohorts [8, 9]. However, the leukocyte population is composed of innate and adaptive immune cells, and to what extent specific immune cell populations are affected by carotenoids is largely under-investigated. Carotenoid supplementation in humans has been shown to increase CD4^+^ T cell proliferation [10] while, on the other hand, a high intake of fruits and vegetables has been linked to lower counts of lymphocytes in a general population cohort [11].

In the present study, we hypothesized that plasma levels of carotenoids associate with certain leukocyte subsets and, furthermore, that these leukocyte subsets may influence the levels of inflammatory markers. The inter-relationships among plasma carotenoids, soluble inflammatory markers, and circulating leukocyte subsets were systematically elucidated by mediation analyses in a large, well-characterized middle-aged population.

## Methods

### Study population

The Swedish CArdioPulmonary bioImage Study (SCAPIS) is a multicentre population-based prospective study comprising 30,000 individuals aged 50–64 recruited between 2013 and 2018 at six Swedish university hospitals [12]. The SCAPIS Leukocyte subcohort of 1078 participants was consecutively recruited from the SCAPIS center at Linköping University Hospital to establish an immune cell profile in whole fresh blood using flow cytometry [13]. Seventy-two subjects were excluded from the study due to active inflammation defined as CRP ≥ 10 mg/L (*n* = 27), technical failure in analyzing plasma carotenoids (*n* = 9), or suspected intake of carotenoid supplements based on exceptionally high levels of plasma carotenoids (*n* = 36). This study uses a cross-sectional design, analyzing data collected at the baseline of the subcohort. A study flow chart is shown in Additional file 1: Fig. S1.

### Plasma carotenoids

Carotenoids (lutein + zeaxanthin, β-cryptoxanthin, lycopene, α-carotene, β-carotene) were quantified using a modified version of a high-performance liquid chromatography (HPLC) method previously described [14]. Lutein and zeaxanthin are presented as a combined value as they are isomers, with zeaxanthin estimated to represent less than 15% of this total [15]. To improve readability, the word “lutein” implies “lutein + zeaxanthin” from this point onwards. See Supplementary Methods for details (Additional file 1: Supplementary methods and Fig. S2).

### Leukocyte subsets

Measurements of leukocyte subsets in EDTA-whole blood were performed within 1 h after blood collection at the Department of Clinical Immunology and Transfusion Medicine, Linköping University Hospital, Linköping, Sweden. BD Multitest Trucount tubes (Becton, Dickinson and Company, Franklin Lakes, USA) were used to obtain absolute counts of granulocytes, monocytes, lymphocytes, natural killer (NK) cells, B cells and T cells, including CD4 + and CD8 + T cells. See Supplementary Methods for details (Additional file 1: Supplementary methods and Fig. S3–4). The absolute count of leukocytes is the sum of the granulocyte, monocyte, and lymphocyte counts.

### Lipid profile, blood glucose, and inflammatory markers

Clinical plasma parameters including total cholesterol, high-density lipoprotein (HDL) cholesterol, low-density lipoprotein (LDL) cholesterol, triglycerides, along with glycated hemoglobin (HbA1c) were determined in fasted plasma samples by the Department of Clinical Chemistry, Linköping University Hospital following manufacturers’ protocols. CRP was measured in plasma using an immunoturbidimetric assay with a Roche Cobas C502 analyzer (Roche Diagnostics, Scandinavia AB) with an inter-assay CV of 2.2%.

IL-18, IL-6, MMP-9, and MPO were quantified in plasma via electrochemiluminescence using the U-Plex Meso Scale Discovery (MSD) platform (Rockville, USA) following manufacturers’ instructions. Analysis was performed at SciLifeLab Affinity Proteomics Unit, Uppsala, Sweden. Inter-assay CVs were 10.6% for IL-18, 21.3% for IL-6, 5.4% for MMP-9, and 11.6% for MPO.

### Assessment of covariates

History of chronic diseases, including cardiovascular diseases, diabetes, cancer, and autoimmune diseases, and use of antihypertensive or cholesterol-lowering medication were self-reported using a questionnaire. Smoking was categorized into active smokers or non-smokers. Nutrient and alcohol intake were estimated using a validated food-frequency questionnaire Meal-Q (MiniMeal-Q) [16].

Moderate and vigorous physical activity patterns were quantified using tri-axial accelerometers (ActiGraph LCC, Pensacola, FL, USA) worn for seven days during waking hours, as previously described [17]. Participants were only included if they had a minimum of 600 min of valid daily wear time on at least four days. Physical activity level was expressed as daily tri-axial vector magnitude counts per minute (cpm). Moderate and vigorous physical activity was defined as periods with ≥ 2690 cpm, presented as a percentage of total wear time.

Body mass index (BMI) was calculated by dividing the body weight in kilograms by the square of height in meters. Weight was measured in light clothing on calibrated scales. Abdominal obesity was defined as a waist-to-hip ratio of ≥ 0.85 for women and ≥ 0.90 for men. Blood pressure was measured in the right brachial artery following a 5-min supine rest. An average of three measurements was used.

### Statistical analyses

Statistical analysis was done using IBM SPSS Statistics (version 28.0). Plasma levels of carotenoids were analyzed alone (lutein, β-cryptoxanthin, lycopene, α-carotene, β-carotene) or combined as total carotenoids. Characteristics across genders and tertiles of carotenoids in plasma were compared using the chi-square test for categorical variables and the Mann–Whitney *U* tests (across genders) or the Kruskal–Wallis *H* test (across tertiles) for continuous variables.

Univariate correlations between tertiles of carotenoids, both leukocyte subsets and inflammatory markers, were determined using Spearman’s *r* correlation coefficient. Leukocyte subsets and inflammatory markers were log-transformed to meet assumptions of linearity. Linear multiple regression was used to further adjust for covariates and to assess the relationship between tertiles of carotenoids and leukocyte subsets or inflammatory markers. Only the leukocyte subsets significantly correlated with carotenoids were analyzed in this step. Regression was run in three separate models; model 0—unadjusted, model 1—adjusted for age and gender, and model 2—adjusted for age, gender, abdominal obesity, total cholesterol, anti-hypertensive medication (self-reported), smoking status, diabetes mellitus (self-reported), estimated intake of polyunsaturated fatty acids, and levels of moderate and vigorous physical activity. Model 2 was referred to as the full adjustment model in the present study. Leukocyte subsets and inflammatory markers were log-transformed to meet assumptions of linearity. The percentage change of leukocyte subsets or inflammatory markers corresponding to each increasing terile of plasma carotenoid levels was obtained following the formula (exp(β)−1*100%, where exp stands for exponentiation. The obtained values were named “effect size.” Two-tailed *p*-values < 0.05 were considered significant.

The associations between different carotenoid levels and various outcomes were further investigated in a series of mediation analyses [18, 19]. All subjects who satisfied the inclusion criteria were included. Mediation analyses hypothesized that the association between the exposure variable (X; plasma carotenoid levels) and the outcome variable (Y) is mediated by a mediator (M). To be qualified for further mediation modeling, significant linear associations (*p* < 0.05) between the following variables had to be found: 1) between the exposure variable (X; plasma carotenoids) and the outcome variable (Y). This is referred to as “XY (Direct effect)” in the present study; 2) between the exposure variable (X; plasma carotenoids) and the mediator variable (M); 3) between the mediator variable (M) and the outcome variable (Y). All parameters were log-transformed to improve linearity. A macro plug-in called PROCESS [18] was used in SPSS to calculate the indirect effect of the exposure mediated by the mediator. This is referred to as “XY (mediated by M)” in the present study. The results were adjusted for the same confounders included in model 2 of the multivariate modeling. Mediation was considered present when the “XY (Direct effect)” was reduced when the mediator was introduced (i.e., “XY (mediated by M)” < “XY (Direct effect)”). The significance of the observed mediation was calculated using the Sobel test [20]. After Bonferroni correction for three-factor comparison, the mediated effects with *p* < 0.017 were considered significant. The variance accounted for (VAF) scores are the ratio of “XY (mediated by M)” by “XY (Direct effect)” which represents the magnitude of the mediation effect. A VAF larger than 0.8 is considered complete mediation. All *p*-values were reported with 3 decimal places. *P*-values less than 0.001 were reported as “ < 0.001”.

Sensitivity analyses were conducted to test the robustness of our main findings. The strategies included [1] modeling the plasma levels of total carotenoid and carotenoid subtypes as continuous variables in the mediation analyses, and [2] conducting multiple imputation of missing values. There was no change in direction or significance of the main findings. In total, 0.5% missing values were spread across 72 individuals in 9 out of 24 variables. No variable had more than 5% missing values. Subjects with missing values for a given variable were excluded from analyses involving that variable.

## Results

### Cohort characteristics

The study population was stratified by total or individual carotenoid levels in plasma. There was a significantly lower proportion of women in the lowest tertiles of total and individual carotenoids, except lycopene (Table 1 and Additional file 1: Table S1–2). Individuals within the lowest tertile of plasma carotenoids also showed lower levels of moderate and vigorous physical activity and were more often smokers. They had higher obesity rates, more antihypertensive medication, higher levels of blood pressure, as well as higher levels of blood glucose and plasma HbA1c. Dyslipidaemia defined by low HDL cholesterol and high triglyceride levels was more prevalent. Furthermore, the prevalence of diabetes and cardiovascular disease was higher in subjects within the lowest tertile. It is worth noting that subjects with high and low plasma lycopene had comparable gender distribution and smoking habits, in contrast to all other carotenoids (Additional file 1: Table S1).

**Table 1:** Characteristics of the study population, including behavioral, physiological, and biochemical variables, plasma carotenoids, immune cell counts, and plasma inflammatory markers

	**Total (*n* = 1006)**	**Total carotenoids**		
		t1 (*n* = 337)	t3 (*n* = 335)	*p*-value
Carotenoid concentration, µmol/L		≤ 1.277	≥ 1.857	
Age	57.2 (53.3–60.9)	58.2 (54.0–61.3)	56.8 (52.8–60.7)	0.046
Females, *n* (%)	481 (47.8)	111 (32.9)	231 (69.0)	< 0.001
**Anthropometry**				
Abdominal obesity ^1^, *n* (%)	667 (66.3)	293 (86.9)	155 (46.3)	< 0.001
BMI, kg m^−2^	26.2 (23.9–29.1)	28.6 (25.8–31.2)	24.5 (22.5–26.9)	< 0.001
BMI ≥ 30 kg m^−2^, *n* (%)	198 (19.7)	120 (35.6)	26 (7.8)	< 0.001
**Lifestyle**				
Moderate and vigorous physical activity, % of wear time	6 (4–8)	5 (3–7)	6 (5–8)	< 0.001
Sedentary physical activity, % of wear time	55 (48–62)	57 (49–63)	54 (46–60)	< 0.001
Alcohol intake, g/day	6.0 (2.4–10.4)	6.4 (2.1–12.4)	4.8 (2.2–8.4)	0.003
Smoker, *n* (%)	73 (7.3)	43 (12.9)	12 (3.6)	< 0.001
**Medication (self-reported)**				
Antihypertensive, n (%)	177 (17.6)	101 (30.0)	26 (7.8)	< 0.001
Cholesterol-lowering, *n* (%)	70 (7.0)	46 (13.6)	3 (0.9)	< 0.001
**Disease status (self-reported)**				
Diabetes, *n* (%)	58 (5.8)	42 (12.7)	8 (2.4)	< 0.001
History of cardiovascular disease ^2^, *n* (%)	28 (2.8)	20 (5.9)	2 (0.6)	< 0.001
Inflammatory disease ^3^, *n* (%)	22 (2.2)	8 (2.4)	10 (3.0)	0.642
Cancer, *n* (%)	59 (5.9)	17 (5.0)	23 (6.9)	0.338
**Blood pressure**				
Systolic, mmHg	129 (120–143)	135 (124–146)	125 (116–139)	< 0.001
Diastolic, mmHg	82 (76–89)	85 (78–91)	80 (74–87)	< 0.001
**Clinical chemistry**				
Total cholesterol, mmol/L	5.4 (4.7–6.1)	5.0 (4.3–5.7)	5.8 (5.0–6.5)	< 0.001
LDL cholesterol, mmol/L	3.2 (2.6–3.8)	3.0 (2.2–3.5)	3.4 (2.8–4.1)	< 0.001
HDL cholesterol, mmol/L	1.6 (1.3–1.9)	1.4 (1.1–1.6)	1.8 (1.5–2.2)	< 0.001
Triglycerides, mmol/L	1.0 (0.8–1.5)	1.3 (1.0–1.9)	0.9 (0.7–1.3)	< 0.001
Glucose, mmol/L	5.6 (5.3–6.0)	5.8 (5.5–6.4)	5.4 (5.1–5.7)	< 0.001
HbA1c, mmol/mol	35 (33–37)	35 (33–39)	34 (32–36)	< 0.001
**Carotenoid levels in plasma**				
Total carotenoids, µmol/L	1.53 (1.13–2.05)	1.00 (0.79–1.14)	2.32 (2.05–2.68)	< 0.001
Lutein, µmol/L	0.27 (0.20–0.36)	0.20 (0.16–0.24)	0.36 (0.30–0.44)	< 0.001
β-cryptoxanthin, µmol/L	0.14 (0.09–0.23)	0.08 (0.06–0.12)	0.26 (0.17–0.36)	< 0.001
Lycopene, µmol/L	0.51 (0.38–0.67)	0.36 (0.27–0.46)	0.67 (0.55–0.81)	< 0.001
α-carotene, µmol/L	0.08 (0.04–0.13)	0.04 (0.03–0.06)	0.15 (0.10–0.21)	< 0.001
β-carotene, µmol/L	0.45 (0.27–0.71)	0.23 (0.16–0.32)	0.81 (0.66–1.07)	< 0.001
**Immune cell counts**				
Leukocytes, ${10}^{6}\text{cells/L}$	5782 (4787–6771)	6116 (5222–7297)	5332 (4530–6245)	< 0.001
Lymphocytes, ${10}^{6}\text{cells/L}$	1783 (1468–2140)	1840 (1512–2176)	1685 (1403–2071)	0.013
Granulocytes, ${10}^{6}\text{cells/L}$	3284 (2616–4056)	3688 (2934–4464)	2916 (2376–3684)	< 0.001
Monocytes, ${10}^{6}\text{cells/L}$	375 (307–471)	410 (334–517)	345 (283–425)	< 0.001
Natural killer cells, % of lymphocytes	12 (8–16)	12 (8–16)	12 (8–16)	0.823
CD3^+^ T cells, % of lymphocytes	77 (72–81)	77 (71–81)	77 (73–81)	0.752
CD4^+^ T cells, % of lymphocytes	50 (44–56)	50 (44–56)	49 (43–56)	0.306
CD8^+^ T cells, % of lymphocytes	24 (19–31)	24 (19–31)	24 (19–31)	0.527
B cells, % of lymphocytes	11 (8–13)	11 (8–13)	11 (8–14)	0.833
**Inflammatory markers**				
CRP, mg/L	0.9 (0.5–1.9)	1.4 (0.6–2.7)	0.7 (0.4–1.3)	< 0.001
IL-6, pg/mL	1.08 (0.76–1.49)	1.15 (0.83–1.56)	1.04 (0.72–1.49)	0.021
IL-18, pg/mL	343 (263–441)	376 (304–482)	308 (238–397)	< 0.001
MPO, ng/mL	102 (78–137)	104 (81–142)	99 (76–132)	0.058
MMP-9, ng/mL	54.7 (41.2–78.0)	56 (43–80)	52 (40–72)	0.008

Tertiles 1 and 3 were compared using the Mann–Whitney *U* test (numerical data) or chi-squared tests (categorical data). Data are given as median (interquartile range) or number (%)

*t1*, tertile 1; *t3*, tertile 3; *BMI*, body mass index; *LDL*, low-density lipoprotein; *HDL*, high-density lipoprotein, *HbA1c*, glycated haemoglobin; *CRP*, C-reactive protein; *IL*, interleukin; *MPO*, myeloperoxidase*; MMP-9*, matrix metalloproteinase-9

^1^Abdominal obesity was defined as having a waist-to-hip ratio ≥ 0.85 (females) or ≥ 0.90 (males)

^2^Previous myocardial infarction, stroke, or stable angina

^3^Rheumatoid arthritis or inflammatory bowel disease, including Crohn’s disease and ulcerative colitis

As shown in Table 1, individuals in the lowest tertile of plasma total carotenoids had significantly higher counts of leukocytes, lymphocytes, granulocytes, and monocytes compared to those in the top tertiles, whereas no differences were observed in T cells, T cell subsets, B cells, or NK cells. Also, the levels of soluble inflammatory markers, except MPO, were higher in those with lower levels of total carotenoids; CRP and IL-18 showed the largest differences. When the cohort was stratified in tertiles of individual carotenoids, similar patterns were observed, except for the differences in lymphocyte counts and IL-6 levels, which became less apparent (Additional file 1: Table S3–4).

Subjects with lower levels of total carotenoids had lower calculated energy intake as well as lower intake of protein, fiber, and fat. They also had lower intake of vitamins and minerals in general, while they consumed more carbohydrates and saturated fatty acids (Additional file 1: Table S5).

### Correlations between plasma carotenoids, leukocyte subsets, and soluble inflammatory markers

**Fig. 1:**
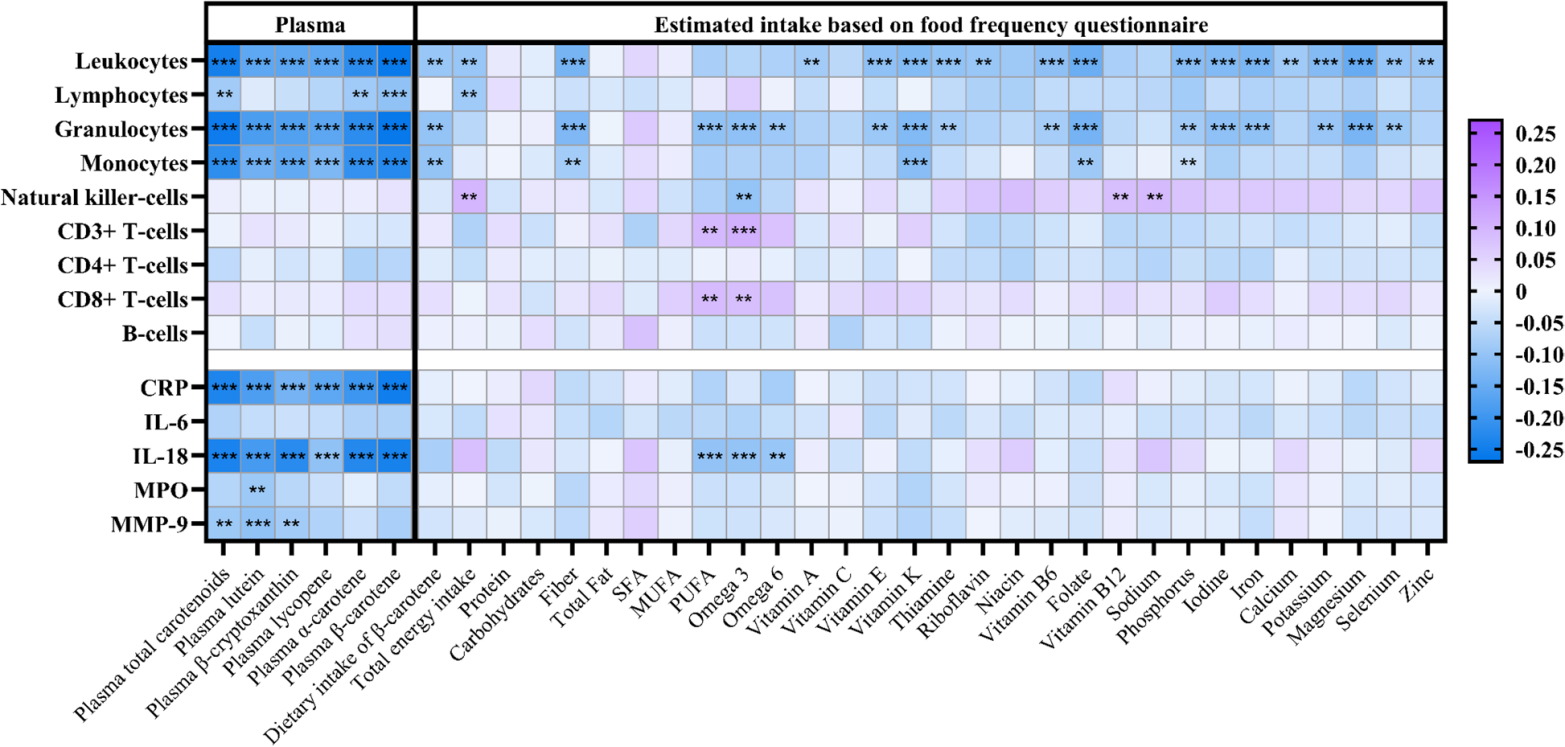
A heatmap indicating the Spearman correlation coefficients between nutritional parameters and counts of leukocyte subtypes and inflammatory markers. Nutritional parameters include plasma carotenoids and estimated dietary intake of macro- and micronutrients derived from food frequency questionnaires. Nutrients that showed no significant correlation with any plasma carotenoid were excluded from this figure. The color gradient reflects the strength and direction of correlations, with blue indicating negative correlations and purple indicating positive correlations. CRP, C-reactive protein; IL, interleukin; MPO, myeloperoxidase; MMP-9, matrix metalloproteinase-9; SFA, saturated fatty acids; MUFA, monounsaturated fatty acids; PUFA, polyunsaturated fatty acids. *** *p* < 0.001; ** *p* < 0.01. *p* < 0.05 not shown

Tertiles of total and individual carotenoids showed univariate correlations with leukocytes, monocytes, and granulocytes (*p* < 0.001) (Fig. 1). Total carotenoids, α- and β-carotene showed weaker but significant associations with lymphocyte counts. However, none of the carotenoids were related to any lymphocyte subsets. Tertiles of total and individual carotenoids also showed univariate correlations to IL-18 and CRP (*p* < 0.001). Total carotenoids, lutein, and β-cryptoxanthin correlated with MMP-9 (*p* < 0.01), while only lutein correlated with MPO (*p* < 0.01). None of the carotenoids were significantly related to IL-6.

### Identification of confounding nutrients

To look for potential confounding nutrients, we analyzed the associations between the estimated intake of main nutrients based on food frequency questionnaires, leukocyte subsets, and inflammatory markers (Fig. 1). Although estimated β-carotene intake was significantly correlated with plasma β-carotene levels (*β* = 0.374, *p* = 0.001), the strength of correlations generated from estimated nutrient intake was generally lower than correlations from plasma carotenoids. Nevertheless, intakes of fiber and most vitamins and minerals showed inverse correlations with counts of leukocytes and granulocytes, likely reflecting that carotenoid-rich fruits and vegetables contain a wide range of vitamins and minerals. However, no significant inverse correlations were observed between soluble inflammatory markers and estimated nutrient intake, except for total polyunsaturated fatty acids (PUFA) and PUFA subclasses omega-3 and omega-6. Calculated intake of PUFAs was therefore included as a confounding nutrient in the multivariate analysis.

### Multivariate adjustment models of plasma carotenoids, leukocyte subsets, and soluble inflammatory markers

**Fig. 2:**
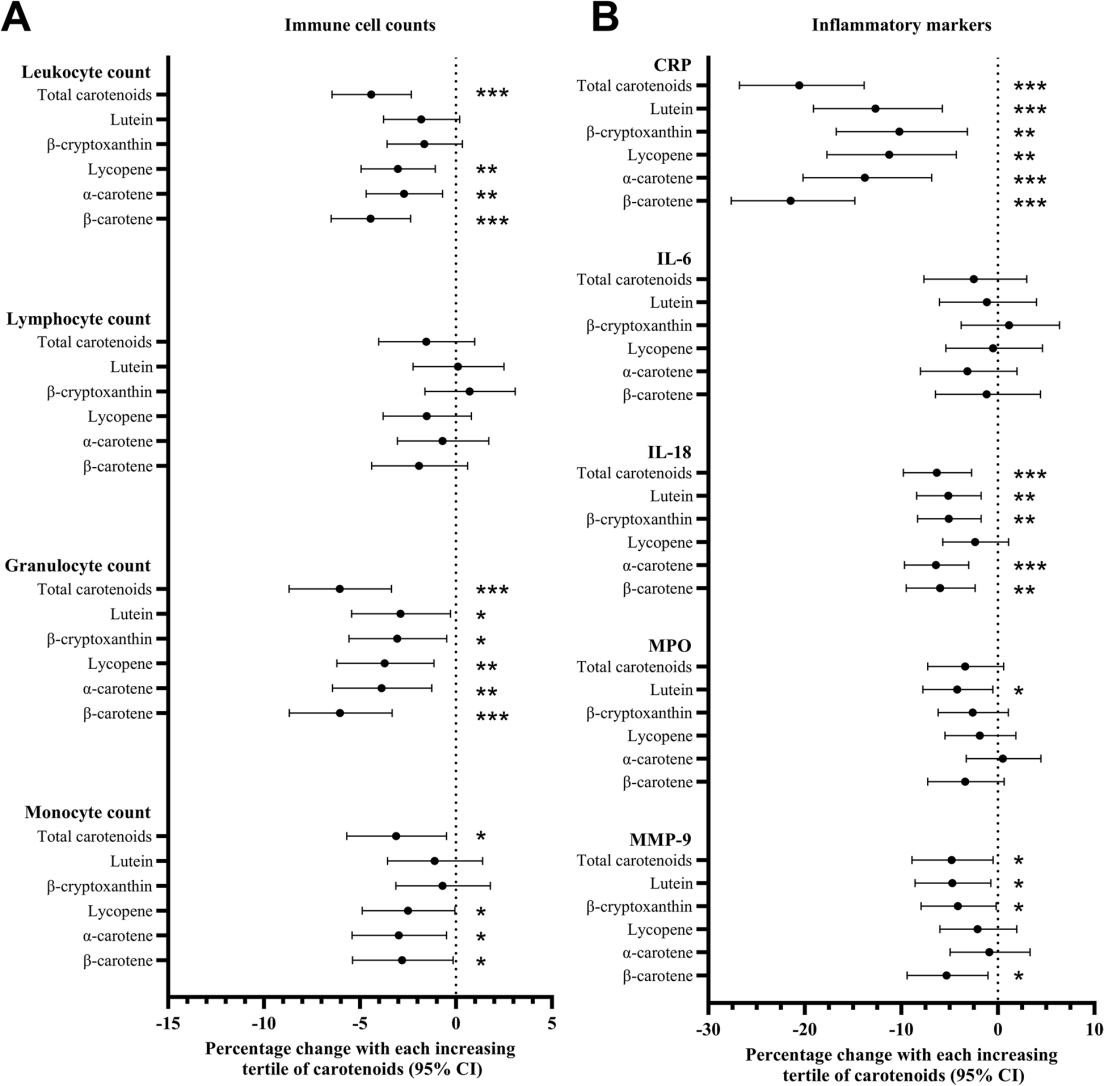
Linear regression models between tertiles of carotenoids and **A** immune cell counts or **B** inflammatory markers. Regression models with inflammatory markers were adjusted for age, gender, total cholesterol, antihypertensive medication, smoking status, abdominal obesity, moderate and vigorous physical activity, self-reported type-2 diabetes, and the proportion of the total daily energy intake from polyunsaturated fat (model 2). Data represents the effect size, which represents the percentage change of the immune cell counts or inflammatory markers with each increasing tertile of carotenoids. All values were log-transformed before regression analysis. Error bars represent the 95% confidence interval. CRP, C-reactive protein; IL, interleukin; MPO, myeloperoxidase; MMP-9, matrix metalloproteinase-9; CI, confidence interval. *** *p* < 0.001; ** *p* < 0.01; * *p* < 0.05

Additional covariates included in the fully adjusted linear regression model were age, gender, abdominal obesity, plasma total cholesterol, anti-hypertensive medication, smoking status, diabetes mellitus, and levels of moderate and vigorous physical activity. After full adjustments, all individual carotenoids remained significantly related to granulocyte counts (Fig. 2A and Additional file 1: Table S6–7). Also, all major carotenoids, except for lutein and β-cryptoxanthin, remained significantly associated with total leukocyte and monocyte counts. β-carotene exhibited the strongest inverse relationship with leukocytes and granulocytes, showing reductions of 4.45% and 6.04% per ascending tertile, respectively. α-carotene showed the strongest inverse relationship with monocyte counts. None of the plasma carotenoids correlated with lymphocyte counts after full adjustment.

The same adjustments were made in linear regression models for soluble inflammatory markers (Fig. 2B and Additional file 1: Table S8–S9). All individual carotenoids remained significantly associated with CRP after full adjustments. The strongest association was seen with total carotenoids and β-carotene; each increasing tertile was associated with a reduction of 20.57% and 21.48%, respectively, in CRP. All individual carotenoids, except lycopene, showed associations with reductions in IL-18 (from 5.08 to 6.41%) with each increasing tertile in full adjustment models. Lutein, β-cryptoxanthin, and β-carotene remained associated with MMP-9 after full adjustments, while only lutein remained associated with MPO. No significant relationships between IL-6 and plasma carotenoids were seen after adjustments.

### Mediation relationships between circulating carotenoids, leukocyte subsets, and inflammatory markers

As shown in Additional file 1: Fig. S5, the counts of leukocytes, granulocytes as well as monocytes correlated with all inflammatory markers. Total lymphocytes, but not lymphocyte subsets, correlated with CRP, MMP-9, and MPO. Collectively, these findings led us to postulate that the relationships between carotenoids and inflammatory markers could be mediated by counts of leukocytes, lymphocytes, granulocytes, and/or monocytes.

**Fig. 3:**
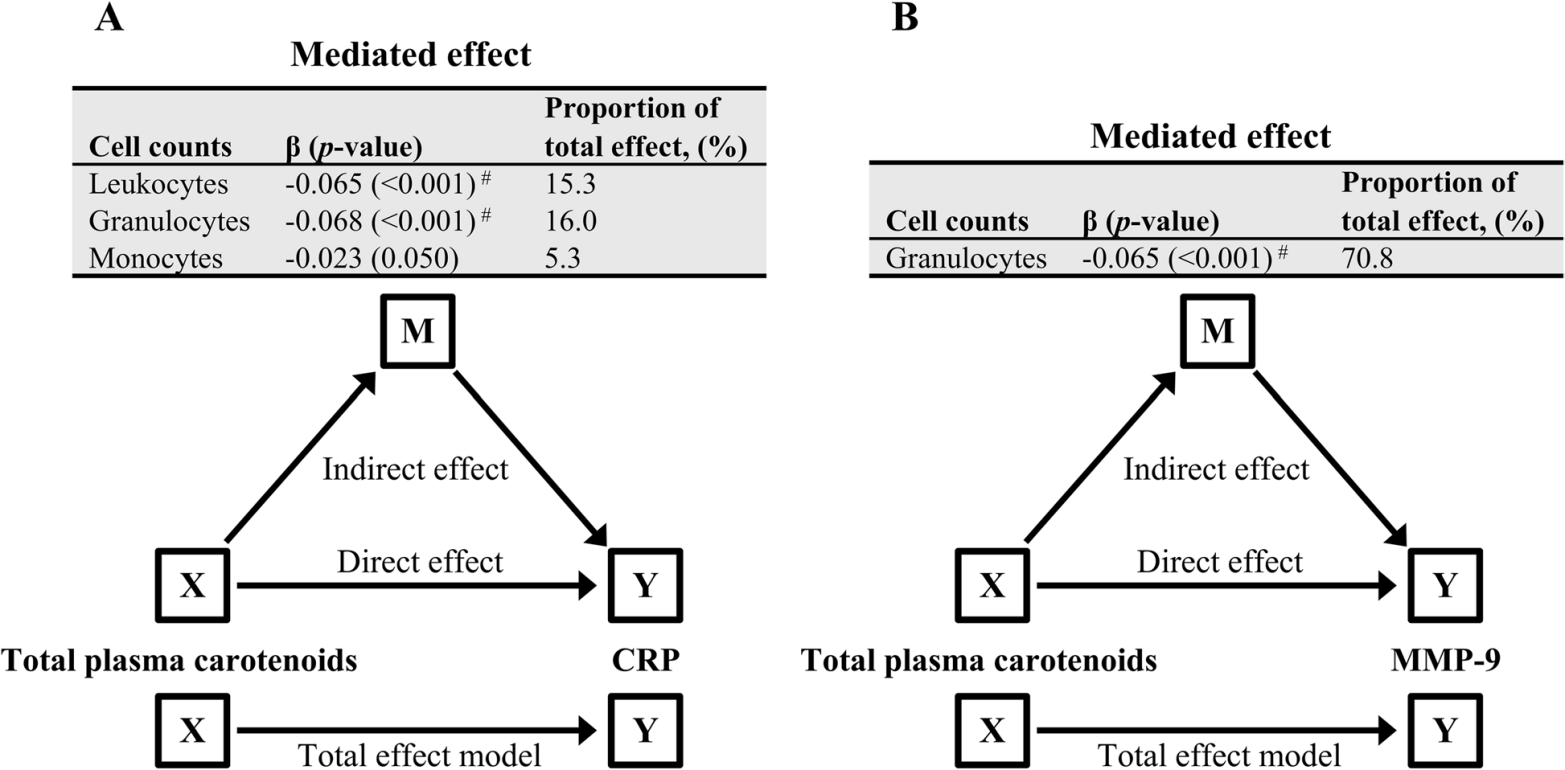
Mediation models. **A** The association between total plasma carotenoids as exposure variable (X), CRP as outcome variable (Y), and cell types that qualified for mediation as mediator variables (M). **B** The association between total plasma carotenoids as exposure variable (X), MMP-9 as outcome variable (Y), and granulocytes as mediator variable (M). All regression analyses were conducted on log-transformed variables. The β-coefficient represents the effect size of the regression model. Significant mediation is denoted by # (Bonferroni corrected threshold *p* < 0.017). CRP, C-reactive protein; MMP-9, matrix metalloproteinase-9

Mediation analyses indicated that the association between total carotenoids and CRP was partially mediated by leukocyte counts, contributing to 15.3% of the effect on CRP. Further dissection of the relationship indicated that this mediation was contributed by granulocytes alone while other cell types either showed insignificant mediation effects or did not qualify to be a mediator (Fig. 3A and Additional file 1: Table S10). Deeper analyses indicated that lutein, α- and β-carotene were the main players in the granulocyte-mediated relationship between plasma carotenoids and CRP (Additional file 1: Table S11).

Furthermore, the mediation analysis showed that the association between total carotenoids and MMP-9 was mediated by granulocyte counts, showing a significant partial mediator effect (70.8% of total effect) (Fig. 3B and Additional file 1: Table S10). Among individual carotenoids, lutein and β-cryptoxanthin both contributed to the granulocyte-mediated relationship with MMP-9 (Additional file 1: Table S11). The significant relationship between MPO and lutein was found to be mediated partially by granulocyte counts, contributing to 46.6% of the total effect.

Regarding the relationship between total carotenoids and IL-18, no significant mediation effect was found by total leukocytes or leukocyte subsets (Additional file 1: Table S10).

## Discussion

In the present study, we comprehensively investigated the inter-relationships among five major carotenoids in plasma, circulating leukocyte subsets, and soluble inflammatory markers in a well-characterized middle-aged population-based cohort. The main findings are that all individual carotenoids were independently associated with one leukocyte subset, namely granulocytes, and that granulocyte counts mediated the inverse correlations between carotenoids and two soluble inflammatory markers, CRP and MMP-9.

To our knowledge, this study is the first to investigate the association between plasma carotenoids and leukocyte subsets in a large population-based cohort. Data from previous comparable cohorts have been limited to total leukocyte counts [8, 9]. One previous study has shown an inverse correlation between plasma β-carotene and neutrophil counts in 40 young male cigarette smokers [21]. Also, in a small cohort of patients with coronary artery disease (*n* = 139), lutein and β-cryptoxanthin levels were inversely associated with neutrophil counts [22]. As neutrophils comprise the vast majority (90%) of granulocytes in the blood, all associations with granulocyte counts in our study were most likely contributed by neutrophils. This assumption was further supported by the findings that total carotenoids and most individual carotenoids were inversely correlated with MMP-9. The association between carotenoids and MMP-9 in plasma has been reported previously in a minor population-based cohort (*n* = 285) [23]. Neutrophils are known to be a major source of MMP-9 in the circulation [24]. Accordingly, mediation analyses showed that the relationships between carotenoids and neutrophil-related granule proteins, i.e., MMP-9 and MPO, were partially mediated by counts of granulocytes, further indicating the interdependent relationships of individual carotenoids with granulocyte counts and neutrophil-associated proteins.

Neutrophils are the most abundant leukocytes in humans and the first responders to acute inflammation. Growing evidence suggests that neutrophils contribute significantly to low-grade chronic inflammation and, in turn, increase the risks of many inflammatory age-related diseases. Elevated neutrophil counts were recently proposed as a causal risk factor for atherosclerotic cardiovascular disease in general populations, while counts of other immune cells did not show similar associations [25, 26]. Elevated neutrophil counts have also been reported to be associated with a negative prognosis in many forms of cancer [27]. Interestingly, the potential of carotenoids to modulate the homeostasis of human neutrophils has been proposed. There are multiple pathways postulated to play a role in the inverse relationship between carotenoids and neutrophil numbers. For example, the breakdown products of carotenoids that are generated under oxidative stress have been shown to promote apoptosis in human neutrophils ex vivo [28].

A few earlier studies have shown that β-carotene supplementation increases the proliferation of CD4^+^ T cells and also increases NK cell activity [10]. A positive correlation between oxygenated carotenoids, such as lutein and β-cryptoxanthin, and circulating NK cells has also been reported in patients with coronary artery disease [22]. However, in this large population-based study, there was no evidence of relationships between plasma carotenoids and counts of lymphocytes or lymphocyte subsets, such as CD4^+^ T cells or NK cells.

A recent clinical trial of IL-6 inhibitor on subjects with chronic low-grade inflammation reported dose-dependent reductions of CRP and neutrophil counts as well as dose-dependent increases in lymphocyte counts [29]. The relationship between carotenoids and CRP has been documented by a large number of population-based studies [30, 31]. Here, we report a mediating role of granulocyte count between plasma carotenoids and CRP. The results emphasize the involvement of the myeloid compartment in chronic low-grade inflammation and also strengthen the hypothesis that carotenoids have a role in this process.

In contrast to CRP and IL-18, the lack of association between carotenoids and IL-6 may be unexpected. The discrepancy is probably explained by the relatively much lower concentrations of IL-6 in plasma, which results in high inter-assay coefficients of variation. Previous population-based studies have also reported inconsistent findings regarding this association between plasma carotenoid levels and IL-6 [30, 32].

Interestingly, carotenoids in plasma were independently and inversely associated with the inflammasome-mediated product IL-18. This result is in line with previous animal models reporting that carotenoids can directly bind and block inflammasome activity and inhibit IL-18 expression [33]. Further studies should be carried out to understand the effects of carotenoids on the human inflammasome. Overall, it is worth noting that no conclusions on the relationships between plasma carotenoids and immune cell function can be drawn from the present results.

One strength of the present study is the combination of absolute measurement of plasma carotenoids and a calculated estimation of nutrient intake using food frequency questionnaires. A number of studies have proposed that anti-inflammatory effects of diets can be attributed to high intake of carotenoid-rich nutrients [34, 35]. However, it is important to quantify plasma carotenoids since the dietary intake of a nutrient may not adequately reflect its levels in the circulation due to factors affecting its homeostasis, such as variations in intestinal absorption [31]. There are also inherent limitations of food frequency questionnaires, providing estimations of varying reliability [16, 36]. This limitation was made apparent by the moderate correlations between estimated β-carotene intake and absolute plasma levels of β-carotene in the present study.

Given the cross-sectional study design, it is not possible to determine a causal relationship between plasma carotenoids and any marker of inflammation. Although the mediation analyses were direction-specific, the cross-sectional study design inherently precludes the confirmation of the directions of the associations presented. Hence, future dietary intervention studies are needed to validate the reported relationships and the directionality of the relationships.

## Conclusions

In conclusion, our findings highlight the role of granulocytes in the association between carotenoids and low-grade inflammation. There is a further need to delineate the underlying mechanism of carotenoids on granulocytes, specifically neutrophils. Future studies should also consider the utility of granulocyte counts to evaluate the anti-inflammatory effects of dietary interventions, in particular carotenoid-rich foods.

## Supplementary Information

Additional file 1: Supplementary methods, Figures S1-5, and Tables S1-11. Figure S1—Flow chart of exclusion and inclusion procedures. Figure S2—Chromatogram of representative plasma carotenoid profile. Figure S3—Gating strategy for absolute cell counts of leukocytes, granulocytes, monocytes, lymphocytes as well as CD3+ , CD4+ , and CD8+ T cells in whole blood. Figure S4—Gating strategy for CD16+ and CD56 + natural killer cells and CD19 + B cells in whole blood. Figure S5 – Heat map indicating correlations between cells and inflammatory markers. Table S1—Behavioral, physiological and biochemical variables across lower (t1) and upper (t3) tertiles of lutein, β-cryptoxanthin, and lycopene in plasma. Table S2—Behavioral, physiological and biochemical variables across lower (t1) and upper (t3) tertiles of α-carotene and β-carotene in plasma. Table S3—Immune cell counts and levels of inflammatory markers between lower (t1) and upper (t3) tertiles of lutein, β-cryptoxanthin, or lycopene in plasma. Table S4—Immune cell counts and levels of inflammatory markers between lower (t1) and upper (t3) tertiles of plasma α-carotene, or β-carotene in plasma. Table S5—Nutritional parameters in participants within lower (t1) and upper (t3) tertiles of total plasma levels of carotenoids. Table S6—Linear regression models between immune cell counts and total carotenoids, lutein, or β-cryptoxanthin in plasma. Table S7—Linear regression models between immune cell counts and lycopene, α-carotene, or β-carotene in plasma. Table S8—Linear regression models between inflammatory markers and total carotenoids, lutein, or β-cryptoxanthin in plasma. Table S9—Linear regression models between inflammatory markers and lycopene, α-carotene, or β-carotene in plasma. Table S10—Mediation analysis between total carotenoids in plasma, inflammatory markers, and immune cell counts. Table S11—Mediation analysis between individual plasma carotenoids, inflammatory markers, and granulocyte count.

## Data Availability

No datasets were generated or analysed during the current study.

## Abbreviations

BMI: Body mass index
CI: Confidence interval
Cpm: Counts per minute
CRP: C-reactive protein
CV: Coefficients of variation
E%: Percentage of daily energy intake
HbA1c: Glycated hemoglobin
HDL: High-density lipoprotein
HPLC: High-performance liquid chromatography
IBD: Inflammatory bowel disease
IL-6: Interleukin-6
IL-18: Interleukin-18
LDL: Low-density lipoprotein
MiniMeal-Q: Food-frequency questionnaire Meal-Q
MMP-9: Matrix metalloproteinase-9
MPO: Myeloperoxidase
MSD: Meso Scale Discovery
MUFA: Monounsaturated fatty acids
NK: Natural killer
NQ: Not qualified
PUFA: Polyunsaturated fatty acids
SCAPIS: Swedish CArdioPulmonary bioImage Study
SFA: Saturated fatty acids
t1: Terile 1
t3: Terile 3
TNF: Tumor necrosis factor
VAF: Variance accounted for
