# Supplementary material for: Plasma carotenoids are inversely correlated with granulocyte counts and soluble inflammatory markers in a middle-aged population: a cross-sectional study with mediation analysis
Source: BMC Med. 2025 Jul 15;23:427. doi: 10.1186/s12916-025-04266-w (PMC12261581; doi:10.1186/s12916-025-04266-w)
Supplement: Supplementary file 1 — Additional file 1: Supplementary methods, Figures S1-5, and Tables S1-11. Figure S1—Flow chart of exclusion and inclusion procedures. Figure S2—Chromatogram of representative plasma carotenoid profile. Figure S3—Gating strategy for absolute cell counts of leukocytes, granulocytes, monocytes, lymphocytes as well as CD3+ , CD4+ , and CD8+ T cells in whole blood. Figure S4—Gating strategy for CD16+ and CD56 + natural killer cells and CD19 + B cells in whole blood. Figure S5 – Heat map indicating correlations between cells and inflammatory markers. Table S1—Behavioral, physiological and biochemical variables across lower (t1) and upper (t3) tertiles of lutein, β-cryptoxanthin, and lycopene in plasma. Table S2—Behavioral, physiological and biochemical variables across lower (t1) and upper (t3) tertiles of α-carotene and β-carotene in plasma. Table S3—Immune cell counts and levels of inflammatory markers between lower (t1) and upper (t3) tertiles of lutein, β-cryptoxanthin, or lycopene in plasma. Table S4—Immune cell counts and levels of inflammatory markers between lower (t1) and upper (t3) tertiles of plasma α-carotene, or β-carotene in plasma. Table S5—Nutritional parameters in participants within lower (t1) and upper (t3) tertiles of total plasma levels of carotenoids. Table S6—Linear regression models between immune cell counts and total carotenoids, lutein, or β-cryptoxanthin in plasma. Table S7—Linear regression models between immune cell counts and lycopene, α-carotene, or β-carotene in plasma. Table S8—Linear regression models between inflammatory markers and total carotenoids, lutein, or β-cryptoxanthin in plasma. Table S9—Linear regression models between inflammatory markers and lycopene, α-carotene, or β-carotene in plasma. Table S10—Mediation analysis between total carotenoids in plasma, inflammatory markers, and immune cell counts. Table S11—Mediation analysis between individual plasma carotenoids, inflammatory markers, and granulocyte count. [file 12916_2025_4266_MOESM1_ESM.pdf]

Additional file 1

Table of Contents

Supplementary methods........2

Supplementary figures........3

Figure S1. Flow chart of exclusion and inclusion procedures........3

Figure S2. Representative chromatogram illustrating the plasma carotenoid profile of a participant........4

Figure S3. Illustrating the gating strategy for determining the absolute cell counts of leukocytes, granulocytes, monocytes, lymphocytes, as well as CD3+, CD4+, and CD8+ T cells in whole blood........5

Figure S4. Illustrating the gating strategy for the CD16+ and CD56+ natural killer cells as well as CD19+ B cells in whole blood........6

Figure S5. A heat map indicating the Spearman correlation coefficients between cells and inflammatory markers....7

Supplementary tables........8

Table S1. Distribution of behavioral, physiological and biochemical variables across lower (t1) and upper (t3) tertiles of lutein, β-cryptoxanthin, and lycopene in plasma........8

Table S2. Distribution of behavioral, physiological and biochemical variables across lower (t1) and upper (t3) tertiles of α-carotene and β-carotene in plasma........9

Table S3. The difference in immune cell counts and levels of inflammatory markers between subjects from lower (t1) and upper (t3) tertiles of lutein, β-cryptoxanthin, or lycopene in plasma........10

Table S4. The difference in immune cell counts and levels of inflammatory markers between subjects from lower (t1) and upper (t3) tertiles of plasma α-carotene, or β-carotene in plasma........11

Table S5. Illustrating levels of nutritional parameters for all participants and in participants within lower (t1) and upper (t3) tertiles of total plasma levels of carotenoids........12

Table S6. Linear regression models between immune cell counts and tertiles of total carotenoids, lutein, or β-cryptoxanthin in plasma........13

Table S7. Linear regression models between immune cell counts and tertiles of lycopene, α-carotene, or β-carotene in plasma........14

Table S8. Linear regression models between levels of inflammatory markers and tertiles of total carotenoids, lutein, or β-cryptoxanthin in plasma........15

Table S9. Linear regression models between levels of inflammatory markers and tertiles of lycopene, α-carotene, or β-carotene in plasma........16

Table S10. Mediation analysis of the association between total carotenoids in plasma as exposure variable (X), different inflammatory markers as outcome variables (Y), and different types of immune cell counts as mediator variables (M)........17

Table S11. Mediation analysis of the association between individual plasma carotenoids as exposure variable (X), different inflammatory markers as outcome variables (Y), and granulocyte count as mediator variables (M)........18

Supplementary methods

Quantification of plasma carotenoids

Carotenoids (lutein + zeaxanthin, β-cryptoxanthin, lycopene, α-carotene, β-carotene) were extracted from plasma using 95% ethanol with 0.1% butylated hydroxytolene and hexane in a 1:1:3.33 v/v ratio. HPLC analysis was performed using a PU 980 HPLC Pump (Jasco Inc., Japan) with a C18-Chromolith® Performance RP-18 endcapped 100-2mm HPLC column (Merck KGaA, Germany) and Jasco MD-2010 Plus Multiwavelength Detector (Jasco Inc., Japan). The flow rate was set at 1.5 mL/min. Carotenoids were detected at 450nm using a spectrophotometric detector (Jasco UV-975, Japan Spectroscopic Company, Japan) and analysed in Clarity software, version 2.6.5 (DataApex, Czech Republic). The method was validated using a reference sample calibrated against an internal standard (SRM 968F) from the National Institute of Standards and Technology. Plasma with relatively high and low carotenoid levels were used as internal controls (Additional file 1: Figure S2). The intra- and interassay coefficients of variation (CV)s were between 3% and 6%. To improve readability, "lutein + zeaxanthin" will be expressed as "lutein" throughout the Appendix.

Absolute counts of leukocyte subsets

The absolute counts of leukocyte subsets were achieved using BD Multitest cocktail CD3 FITC/CD8 PE/CD45 PerCP/CD4 APC and cocktail CD3 FITC/CD16+CD56 PE/CD45 PerCP/CD19 APC with Trucount tubes containing an exact number of lyophilized beads (Becton, Dickinson and Company, Franklin Lakes, US) were used according to the manufacturer's instructions. Samples were analyzed with a 3-laser BD FACSCanto II (488nm, 633nm, 405nm) and BD FACSDiva Software, version 8.0.1, and the acquisition was stopped when 10,000 cells were collected in the lymphocyte gate.

Two separate Trucount tubes were performed per subject. In tube 1, CD3+, CD4+ and CD8+ T cells were identified (Additional file 1: Figure S3). CD4+ and CD8+ T cells were defined as CD3+CD4+ and CD3+CD8+ respectively. In Tube 2, natural killer (NK) and B cells were identified and defined as CD3- CD16/56+ and CD3-CD19+ respectively (Figure S4). Leukocytes are identified as CD45+ cells in a SSC/CD45 dot plot, and granulocytes, monocytes, lymphocytes, and beads are also identified in this plot according to their respective characteristics. The final absolute count of leukocytes, granulocytes, monocytes, and lymphocytes for each subject was obtained by averaging counts from both Trucount tubes.

Assessment of covariates

To identify confounding nutrients, estimated nutrient intake parameters that showed no significant correlation with any plasma carotenoid were excluded from further analyses. Spearman correlation analyses were carried out between the remaining estimated nutrient intake parameters and all cellular and soluble inflammatory markers.

Supplementary Figures

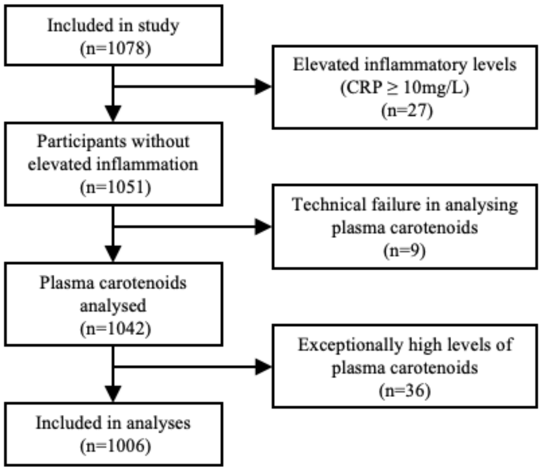

Figure S1. Flow chart of exclusion and inclusion procedures. CRP, C-reactive protein.

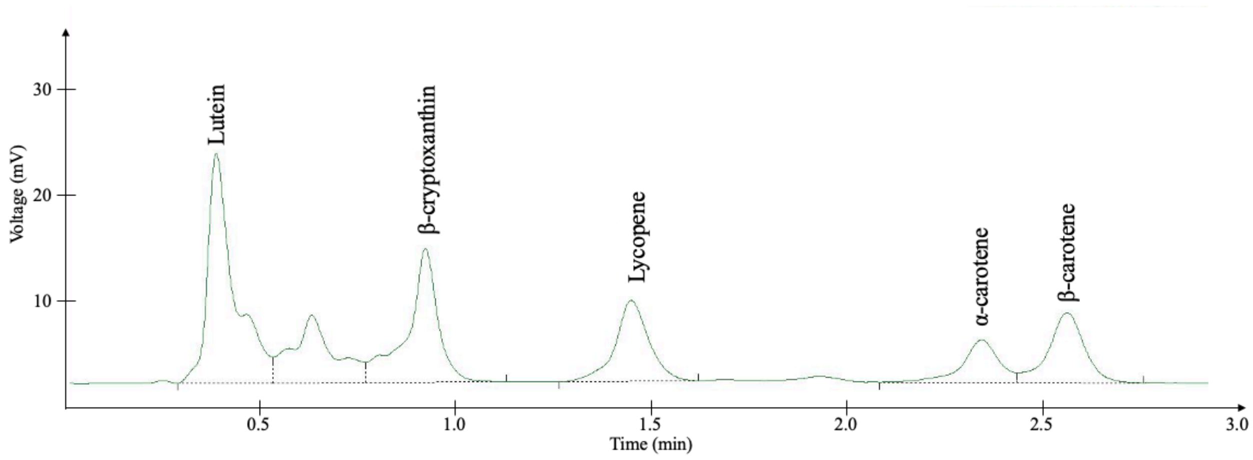

Figure S2. Representative chromatogram illustrating the plasma carotenoid profile of a participant.

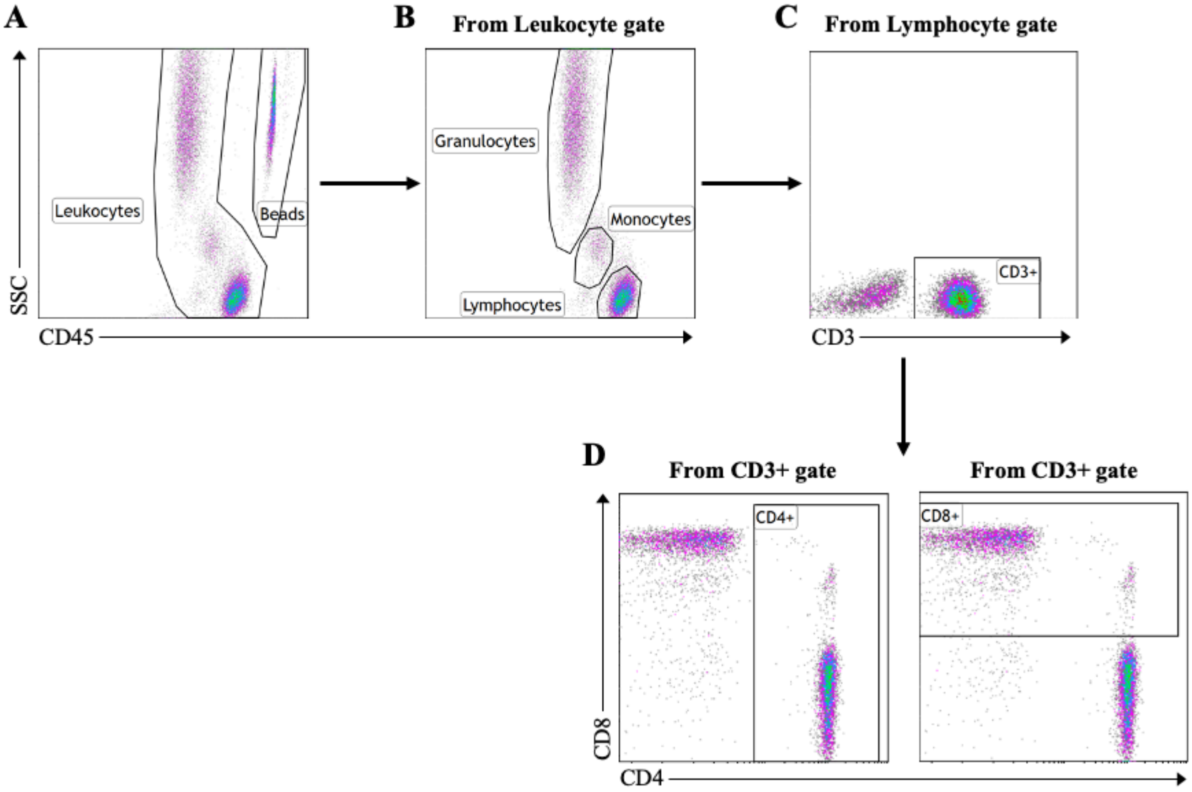

Figure S3. Illustrating the gating strategy for determining the absolute cell counts of leukocytes, granulocytes, monocytes, lymphocytes, as well as CD3+, CD4+, and CD8+ T cells in whole blood. A. Viable leukocytes and Trucount beads were identified in separate gates based on locations in the plot that represent granularity and CD45 expression. B. From the “Leukocyte” gate, granulocytes, monocytes, and lymphocytes were identified and gated based on their expression of CD45 as well as granularity. C. From the “Lymphocyte” gate, CD3+ lymphocytes were identified and gated based on their expression of CD3. D. From the “CD3+” gate, CD4+ and CD8+ T cells were identified and gated based on their expression of CD4 or CD8, respectively.

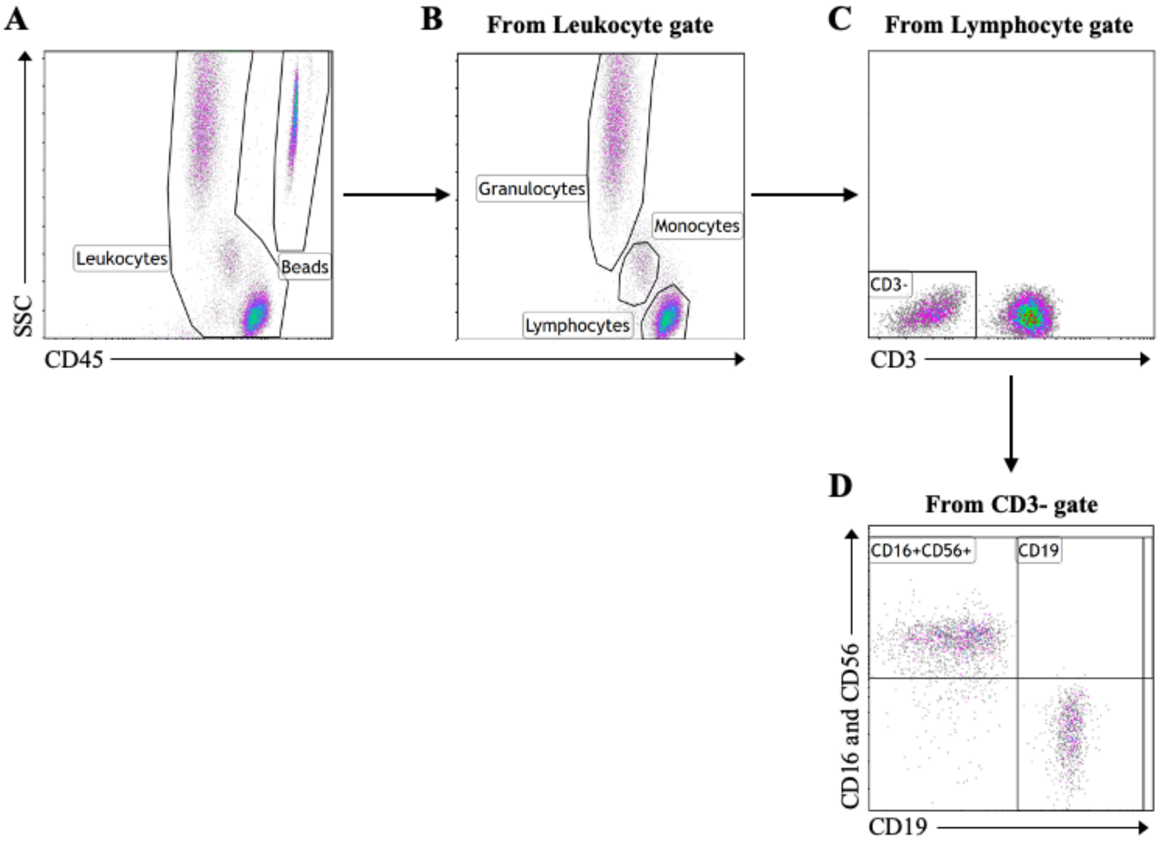

Figure S4. Illustrating the gating strategy for the CD16+ and CD56+ natural killer-cells as well as CD19+ B cells in whole blood. A. Viable leukocytes and Trucount beads were identified in separate gates based on locations in a plot that represents granularity and CD45 expression. B. From the "Leukocyte" gate, granulocytes, monocytes, and lymphocytes were identified and gated based on their expression of CD45 as well as granularity. C. From the "Lymphocyte" gate, CD3- lymphocytes were identified and gated based on their expression of CD3. D. From the "CD3-" gate, CD16+ and CD56+ natural killer-cells, as well as CD19+ B cells, were identified and gated based on their expression of CD16 in combination with CD56 or CD19, respectively.

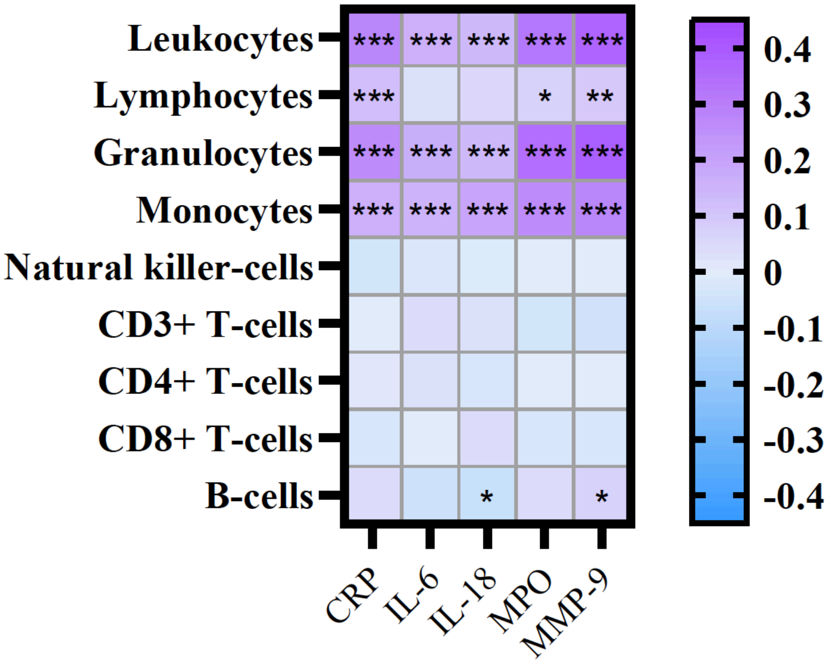

Figure S5. A heat map indicating the Spearman correlation coefficients between cells and inflammatory markers. The color gradient reflects the strength and direction of correlations, with blue indicating negative correlations and purple indicating positive correlations. CRP, C-reactive protein; IL, interleukin; MPO, myeloperoxidase; MMP-9, matrix metalloproteinase-9. *** p<0.001; ** p<0.01, * p<0.05.

Supplementary Tables

Table S1. Distribution of behavioral, physiological, and biochemical variables across lower (t1) and upper (t3) tertiles of lutein, β-cryptoxanthin, and lycopene in plasma.

	**Lutein**			**β-cryptoxanthin**			**Lycopene**		
	t1 (n=336)	t3 (n=332)	*p*-value	t1 (n=339)	t3 (n=335)	*p*-value	t1 (n=337)	t3 (n=334)	*p*-value
Plasma concentration, µmol/L	≤0.224	≥0.327		≤0.108	≥0.195		≤0.428	≥0.613	
Age, years	57.5 (53.4-61.3)	57.3 (53.5-60.9)	0.639	57.8 (53.7-61.2)	57.4 (53.1-61.3)	0.456	58.3 (54.2-61.5)	56.7 (52.4-60.4)	<0.001
Females, n(%)	132 (39.3)	189 (56.9)	<0.001	123 (36.3)	216 (64.5)	<0.001	149 (44.2)	168 (50.3)	0.114
**Anthropometry**									
Abdominal obesity[1], n(%)	263 (78.3)	180 (54.2)	<0.001	268 (79.1)	171 (51.0)	<0.001	255 (75.7)	186 (55.7)	<0.001
BMI, kg m^{-2}	27.7 (24.9-30.8)	25.0 (22.7-26.8)	<0.001	27.7 (24.9-30.6)	25.1 (23.1-27.3)	<0.001	27.7 (24.7-30.5)	25.3 (22.9-27.9)	<0.001
BMI, ≥30 kg m^{-2}, n(%)	104 (31.0)	21 (6.3)	<0.001	105 (31.0)	32 (9.6)	<0.001	101 (30.0)	38 (11.4)	<0.001
**Lifestyle**									
Moderate and vigorous physical activity, % of wear time	5 (4-7)	6 (4-8)	0.001	5 (4-7)	6 (4-8)	0.001	5 (4-7)	6 (4-8)	<0.001
Sedentary physical activity, % of wear time	56 (49-63)	54 (47-60)	0.019	56 (49-63)	54 (47-60)	<0.001	56 (49-63)	54 (46-60)	0.003
Alcohol intake, g/day	6.2 (2.0-11.3)	5.6 (2.8-9.8)	0.963	6.7 (2.9-13.2)	4.9 (2.1-8.4)	<0.001	6.0 (1.9-10.4)	6.4 (2.7-10.5)	0.227
Smokers, n(%)	38 (11.4)	12 (3.6)	<0.001	38 (11.3)	11 (3.3)	<0.001	32 (9.6)	19 (5.7)	0.063
**Medication (self-reported)**									
Antihypertensive, n(%)	84 (25.0)	34 (10.3)	<0.001	89 (26.3)	37 (11.0)	<0.001	87 (25.8)	36 (10.8)	<0.001
Cholesterol-lowering, n(%)	43 (12.8)	8 (2.4)	<0.001	46 (13.6)	10 (3.0)	<0.001	41 (12.2)	10 (3.0)	<0.001
**Disease status (self-reported)**									
Diabetes, n(%)	30 (9.1)	9 (2.7)	<0.001	36 (10.7)	8 (2.4)	<0.001	37 (11.1)	9 (2.7)	<0.001
History of cardiovascular disease[2], n(%)	19 (5.7)	3 (0.9)	<0.001	12 (3.5)	4 (1.2)	0.045	18 (5.3)	3 (0.9)	<0.001
Inflammatory diseases[3], n(%)	9 (2.7)	7 (2.1)	0.617	6 (1.8)	6 (1.8)	0.988	10 (3.0)	7 (2.1)	0.457
Cancer, n(%)	18 (5.4)	21 (6.3)	0.614	16 (4.7)	24 (7.2)	0.182	17 (5.0)	21 (6.3)	0.510
**Blood pressure**									
Systolic, mmHg	133 (123-145)	125 (116-137)	<0.001	132 (121-145)	126 (117-140)	<0.001	133 (122-144)	127 (116-141)	<0.001
Diastolic, mmHg	85 (79-91)	79 (74-86)	<0.001	83 (77-91)	81 (75-87)	<0.001	84 (78-90)	81 (75-88)	0.005
**Clinical chemistry**									
Total cholesterol, mmol/L	5.1 (4.3-5.8)	5.7 (5.0-6.5)	<0.001	5.0 (4.4-5.7)	5.6 (5.0-6.3)	<0.001	5.0 (4.3-5.7)	5.8 (5.1-6.5)	<0.001
LDL cholesterol, mmol/L	3.0 (2.4-3.6)	3.4 (2.8-4.1)	<0.001	3.0 (2.3-3.6)	3.4 (2.7-4.0)	<0.001	3.0 (2.3-3.5)	3.5 (2.9-4.1)	<0.001
HDL cholesterol, mmol/L	1.4 (1.2-1.7)	1.8 (1.5-2.2)	<0.001	1.5 (1.2-1.8)	1.8 (1.4-2.1)	<0.001	1.4 (1.2-1.8)	1.7 (1.4-2.1)	<0.001
Triglycerides, mmol/L	1.2 (0.9-1.6)	0.9 (0.7-1.3)	<0.001	1.2 (0.8-1.6)	0.9 (0.7-1.3)	<0.001	1.2 (0.8-1.6)	1.0 (0.8-1.4)	<0.001
Glucose, mmol/L	5.8 (5.4-6.2)	5.5 (5.2-5.8)	<0.001	5.8 (5.4-6.3)	5.5 (5.2-5.8)	<0.001	5.7 (5.3-6.2)	5.6 (5.2-5.9)	<0.001
HbA1c, mmol/mol	35 (33-38)	35 (32-36)	<0.001	35 (33-38)	35 (33-36)	0.004	35 (33-39)	34 (32-36)	<0.001

Tertile 1 and 3 were compared using the Mann-Whitney U (numerical data) or chi-squared-tests (categorical data). Data are given as median (interquartile range) or number (%).

[1] Abdominal obesity was defined as having a waist-to-hip ratio ≥0.85 (females) or ≥0.90 (males).

[2] Previous myocardial infarction, stroke, or stable angina.

[3] Rheumatoid arthritis or inflammatory bowel disease, including Crohn´s disease and ulcerative colitis.

t1, tertile 1; t3, tertile 3; BMI, body mass index; IBD, inflammatory bowel disease; LDL, low-density lipoprotein HDL, high-density lipoprotein, HbA1c, glycated haemoglobin.

Table S2. Distribution of behavioral, physiological, and biochemical variables across lower (t1) and upper (t3) tertiles of α-carotene and β-carotene in plasma.

	**α-carotene**			**β-carotene**		
	t1 (n=339)	t3 (n=334)	*p*-value	t1 (n=336)	t3 (n=335)	*p*-value
Plasma concentration, µmol/L	≤0.055	≥0.105		≤0.326	≥0.611	
Age	58.2 (54.0-61.4)	56.8 (52.9-60.9)	0.065	57.8 (53.8-61.1)	56.9 (52.8-60.6)	0.134
Females, n(%)	115 (33.9)	217 (65.0)	<0.001	98 (29.2)	239 (71.3)	<0.001
**Anthropometry**						
Abdominal obesity[1], n(%)	281 (82.9)	167 (50.0)	<0.001	295 (87.8)	144 (43.0)	<0.001
BMI, kg m^{-2}	28.0 (25.6-31.0)	24.7 (22.6-27.3)	<0.001	28.4 (25.6-31.0)	24.5 (22.6-26.9)	<0.001
BMI ≥30 kg m^{-2}, n(%)	112 (33.0)	30 (9.0)	<0.001	117 (34.8)	32 (9.6)	<0.001
**Lifestyle**						
Moderate and vigorous physical activity, % of wear time	5 (4-7)	6 (4-8)	<0.001	5 (4-7)	6 (5-8)	<0.001
Sedentary physical activity, % of wear time	56 (49-63)	54 (46-60)	<0.001	57 (49-65)	53 (47-59)	<0.001
Alcohol intake, g/day	7.2 (2.7-13.3)	4.7 (2.3-8.2)	<0.001	7.5 (2.9-14.3)	4.8 (2.1-8.1)	<0.001
Smokers, n(%)	45 (13.4)	9 (2.7)	<0.001	40 (12.0)	11 (3.3)	<0.001
**Medication (self-reported)**						
Antihypertensive, n(%)	97 (28.6)	36 (10.8)	<0.001	99 (29.6)	35 (10.4)	<0.001
Cholesterol-lowering, n(%)	48 (14.2)	8 (2.4)	<0.001	51 (15.2)	7 (2.1)	<0.001
**Disease status (self-reported)**						
Diabetes, n(%)	40 (11.9)	7 (2.1)	<0.001	42 (12.7)	4 (1.2)	<0.001
History of cardiovascular disease[2], n(%)	22 (6.5)	2 (0.6)	<0.001	19 (5.7)	4 (1.2)	0.001
Inflammatory disease[3], n(%)	10 (2.9)	8 (2.4)	0.656	7 (2.1)	7 (2.1)	0.991
Cancer, n(%)	13 (3.8)	25 (7.5)	0.040	14 (4.2)	26 (7.8)	0.053
**Blood pressure**						
Systolic, mmHg	135 (123-145)	126 (116-139)	<0.001	135 (123-146)	125 (116-141)	<0.001
Diastolic, mmHg	85 (78-91)	80 (75-86)	<0.001	86 (78-92)	81 (74-87)	<0.001
**Clinical chemistry**						
Total cholesterol, mmol/L	5.2 (4.4-5.9)	5.6 (4.9-6.3)	<0.001	5.2 (4.4-5.9)	5.6 (4.9-6.3)	<0.001
LDL cholesterol, mmol/L	3.1 (2.3-3.7)	3.3 (2.7-4.0)	<0.001	3.0 (2.3-3.6)	3.3 (2.7-4.0)	<0.001
HDL cholesterol, mmol/L	1.4 (1.1-1.7)	1.8 (1.4-2.1)	<0.001	1.3 (1.1-1.6)	1.8 (1.5-2.2)	<0.001
Triglycerides, mmol/L	1.3 (0.9-1.8)	0.9 (0.7-1.2)	<0.001	1.4 (1.0-1.9)	0.9 (0.7-1.2)	<0.001
Glucose, mmol/L	5.8 (5.5-6.4)	5.4 (5.1-5.8)	<0.001	5.9 (5.5-6.5)	5.4 (5.1-5.7)	<0.001
HbA1c, mmol/mol	36 (33-39)	35 (33-36)	<0.001	35 (33-39)	34 (32-36)	<0.001

Tertile 1 and 3 were compared using the Mann-Whitney U (numerical data) or chi-squared-tests (categorical data). Data are given as median (interquartile range) or number (%).

[1] Abdominal obesity was defined as having a waist-to-hip ratio ≥0.85 (females) or ≥0.90 (males).

[2] Previous myocardial infarction, stroke, or stable angina.

[3] Rheumatoid arthritis or inflammatory bowel disease, including Crohn´s disease and ulcerative colitis.

t1, tertile 1; t3, tertile 3; BMI, body mass index; IBD, inflammatory bowel disease; LDL, low-density lipoprotein HDL, high-density lipoprotein, HbA1c, glycated haemoglobin.

Table S3. The difference in immune cell counts and levels of inflammatory markers between subjects from lower (t1) and upper (t3) tertiles of lutein, β-cryptoxanthin, or lycopene in plasma.

	Lutein			β-cryptoxanthin			Lycopene		
	t1 (n=336)	t3 (n=332)	*p*-value	t1 (n=339)	t3 (n=335)	*p*-value	t1 (n=337)	t3 (n=334)	*p*-value
Plasma concentration, μmol/L	≤0.224	≥0.327		≤0.108	≥0.195		≤0.428	≥0.613	
**Immune cell counts**									
Leukocytes, $\times 10^6$cells/L	5950 (5019-7048)	5525 (4663-6296)	<0.001	5998 (5148-7127)	5539 (4658-6526)	<0.001	6109 (5209-7056)	5486 (4647-6422)	<0.001
Lymphocytes $\times 10^6$cells/L	1794 (1503-2164)	1779 (1461-2111)	0.418	1804 (1493-2147)	1752 (1446-2114)	0.405	1846 (1526-2202)	1751 (1409-2106)	0.030
Granulocytes, $\times 10^6$cells/L	3493 (2733-4309)	3074 (2449-3761)	<0.001	3511 (2844-4398)	3136 (2514-3904)	<0.001	3580 (2792-4322)	3064 (2481-3742)	<0.001
Monocytes, $\times 10^6$cells/L	391 (319-497)	359 (293-440)	<0.001	392 (325-502)	361 (286-446)	<0.001	403 (327-512)	362 (294-447)	<0.001
Natural killer cells, % of lymphocytes	12 (8-16)	11 (8-15)	0.702	12 (8-16)	12 (8-15)	0.969	12 (8-16)	12 (8-16)	0.500
$CD3^+$ T cells, % of lymphocytes	77 (71-82)	78 (72-81)	0.320	77 (71-82)	77 (73-81)	0.656	76 (71-82)	76 (72-81)	0.911
$CD4^+$ T cells, % of lymphocytes	50 (45-56)	50 (43-56)	0.978	50 (43-56)	49 (43-56)	0.626	50 (44-56)	50 (43-56)	0.587
$CD8^+$ T cells, % of lymphocytes	24 (18-31)	24 (19-31)	0.919	25 (19-32)	24 (19-31)	0.863	24 (18-31)	23 (19-30)	0.615
B cells, % of lymphocytes	11 (8-14)	10 (8-13)	0.169	10 (8-13)	10 (8-13)	0.995	11 (8-14)	11 (8-13)	0.511
**Inflammatory markers**									
CRP, mg/L	1.2 (0.6-2.3)	0.8 (0.4-1.3)	<0.001	1.0 (0.6-2.3)	0.8 (0.4-1.6)	<0.001	1.2 (0.6-2.6)	0.8 (0.4-1.5)	<0.001
IL-6, pg/mL	1.14 (0.81-1.55)	1.03 (0.73-1.44)	0.076	1.09 (0.77-1.49)	1.03 (0.73-1.49)	0.582	1.13 (0.81-1.53)	1.05 (0.72-1.47)	0.116
IL-18, pg/mL	375 (293-465)	309 (243-407)	<0.001	376 (297-486)	312 (242-402)	<0.001	362 (290-472)	328 (250-429)	0.004
MPO, ng/mL	104 (81-145)	99 (74-136)	0.023	105 (80-142)	101 (77-133)	0.121	105 (82-143)	102 (76-138)	0.154
MMP-9, ng/mL	58 (43-79)	51 (39-77)	0.006	57 (43-85)	52 (40-76)	0.019	59 (44-79)	53 (40-78)	0.024

Tertile 1 and 3 were compared using the Mann-Whitney U-test. Data are given as median (interquartile range).
t1, tertile 1; t3, tertile 3; CRP, C-reactive protein; IL, Interleukin; MPO, Myeloperoxidase; MMP-9, Matrix metalloproteinase-9.

Table S4. The difference in immune cell counts and levels of inflammatory markers between subjects from lower (t1) and upper (t3) tertiles of plasma α-carotene, or β-carotene in plasma.

	**α-carotene**			**β-carotene**		
	t1 (n=339)	t3 (n=334)	*p*-value	t1 (n=336)	t3 (n=335)	*p*-value
Plasma concentration, µmol/L	≤0.055	≥0.105		≤0.326	≥0.611	
**Immune cell counts**						
Leukocytes, $\times 10^6$cells/L	6079 (5209-7183)	5404 (4528-6336)	<0.001	6167 (5198-7288)	5311 (4453-6257)	<0.001
Lymphocytes $\times 10^6$cells/L	1840 (1507-2220)	1706 (1403-2101)	0.025	1842 (1524-2203)	1701 (1395-2067)	0.003
Granulocytes, $\times 10^6$cells/L	3547 (2861-4327)	3009 (2375-3727)	<0.001	3546 (2877-4520)	2898 (2357-3687)	<0.001
Monocytes, $\times 10^6$cells/L	408 (333-506)	344 (286-425)	<0.001	413 (334-512)	345 (283-418)	<0.001
Natural killer cells, % of lymphocytes	12 (8-16)	12 (8-16)	0.918	12 (8-16)	12 (9-16)	0.468
$CD3^+$ T cells, % of lymphocytes	77 (72-82)	77 (73-81)	0.626	77 (71-81)	76 (72-81)	0.381
$CD4^+$ T cells, % of lymphocytes	51 (45-56)	49 (43-56)	0.025	50 (45-56)	50 (43-56)	0.149
$CD8^+$ T cells, % of lymphocytes	24 (19-31)	25 (19-32)	0.217	24 (18-31)	24 (20-31)	0.313
B cells, % of lymphocytes	10 (8-13)	10 (8-13)	0.630	10 (8-13)	11 (8-14)	0.218
**Inflammatory markers**						
CRP, mg/L	1.4 (0.7-2.7)	0.8 (0.4-1.4)	<0.001	1.3 (0.7-2.3)	0.8 (0.4-1.5)	<0.001
IL-6, pg/mL	1.12 (0.83-1.54)	1.03 (0.74-1.49)	0.021	1.13 (0.83-1.56)	1.01 (0.73-1.48)	0.069
IL-18, pg/mL	381 (308-486)	310 (243-397)	<0.001	388 (310-486)	312 (242-413)	<0.001
MPO, ng/mL	103 (80-139)	99 (75-130)	0.722	102 (77-133)	100 (77-133)	0.104
MMP-9, ng/mL	56 (43-79)	52 (40-72)	0.355	54 (42-78)	53 (41-77)	0.011

Tertile 1 and 3 were compared using the Mann-Whitney U-test. Data are given as median (interquartile range).

t1, tertile 1; t3, tertile 3; CRP, C-reactive protein; IL, Interleukin; MPO, Myeloperoxidase; MMP-9, Matrix metalloproteinase-9.

Table S5. Illustrating levels of nutritional parameters for all participants and in participants within lower (t1) and upper (t3) tertiles of total plasma levels of carotenoids. Nutritional parameters include estimated dietary intake of macro- and micronutrients derived from food frequency questionnaires.

	**Total (n=1006)**	**Total carotenoids**		
		t1 (n=337)	t3 (n=335)	*p*-value
Plasma concentration, µmol/L		≤1.277	≥1.857	
Total energy intake, kcal/day	1582 (1254-1991)	1581 (1217-2004)	1649 (1316-2032)	0.148
**Macronutrients**				
Protein, E%	16.1 (14.4-17.8)	15.6 (14.0-17.6)	16.2 (14.6-17.9)	0.011
Carbohydrates, E%	42.9 (38.7-46.5)	43.6 (39.3-47.9)	42.2 (38.5-45.6)	0.003
Fiber, g/day	17.5 (12.4-23.7)	15.1 (10.2-21.0)	20.1 (14.5-25.9)	<0.001
Fat, E%	35.7 (32.3-39.3)	35.0 (31.9-38.6)	37.1 (33.1-39.9)	<0.001
SFA, E%	13.8 (12.1-15.6)	14.2 (12.4-16.4)	13.7 (12.1-15.4)	0.009
MUFA, E%	13.0 (11.5-14.5)	12.6 (11.3-14.0)	13.4 (11.9-15.0)	<0.001
PUFA, E%	5.3 (4.1-7.0)	4.7 (3.7-5.9)	6.1 (4.8-8.0)	<0.001
Omega 3, E%	1.1 (0.8-1.3)	1.0 (0.7-1.2)	1.1 (0.9-1.6)	<0.001
Omega 6, E%	4.0 (3.1-5.3)	3.4 (2.8-4.5)	4.5 (3.6-6.0)	<0.001
**Vitamins**				
Vitamin A, µg/day	649 (453-865)	576 (399-797)	719 (543-978)	<0.001
Retinol, µg/day	286 (207-396)	292 (209-408)	294 (218-395)	0.756
Vitamin C, mg/day	73 (49-104)	62 (40-92)	87 (57-120)	<0.001
Vitamin D, µg/day	5.6 (4.0-7.7)	5.4 (3.8-8.0)	5.7 (4.3-7.5)	0.772
Vitamin E, mg/day	8.4 (6.4-11.3)	7.5 (5.7-10.0)	9.5 (7.4-12.3)	<0.001
Vitamin K, µg/day	26 (18-36)	22 (15-31)	30 (22-42)	<0.001
Thiamine, mg/day	1.1 (0.9-1.5)	1.1 (0.8-1.4)	1.3 (1.0-1.7)	<0.001
Riboflavin, mg/day	1.5 (1.1-1.9)	1.4 (1.0-1.9)	1.6 (1.2-2.0)	0.001
Niacin, niacinequivalents/day	29 (24-35)	29 (23-35)	30 (25-36)	0.034
Vitamin B6, mg/day	1.6 (1.3-2.0)	1.5 (1.1-1.9)	1.7 (1.3-2.1)	<0.001
Folate, µg/day	289 (222-366)	256 (192-336)	327 (255-395)	<0.001
Vitamin B12, µg/day	4.0 (2.9-5.2)	3.9 (2.7-5.2)	4.2 (3.3-5.3)	0.030
**Minerals**				
Sodium, g/day	2.1 (1.6-2.6)	2.1 (1.6-2.6)	2.1 (1.7-2.7)	0.069
Phosphorus, mg/day	1255 (983-1591)	1225 (913-1590)	1357 (1087-1629)	<0.001
Iodine, µg/day	86 (61-127)	83 (56-116)	92 (66-138)	0.001
Iron, mg/day	9.5 (7.1-12.5)	8.4 (6.3-11.7)	10.4 (7.9-13.6)	<0.001
Calcium, mg/day	839 (619-1119)	833 (592-1140)	905 (702-1130)	0.033
Potassium, mg/day	2887 (2323-3500)	2735 (2185-3394)	3062 (2516-3691)	<0.001
Magnesium, mg/day	320 (252-404)	294 (229-371)	353 (287-442)	<0.001
Selenium, µg/day	41 (30-58)	36 (27-47)	47 (33-68)	<0.001
Zinc, mg/day	8.8 (7.0-11.0)	8.4 (6.7-10.8)	9.4 (7.5-11.4)	<0.001

Tertile 1 and 3 were compared using the Mann-Whitney U test. Data are given as median (interquartile range). t1, tertile 1; t3, tertile 3; E%, percentage of daily energy intake; SFA, saturated fatty acids; MUFA, monounsaturated fatty acids; PUFA, polyunsaturated fatty acids.

Table S6. Linear regression models between immune cell counts and tertiles of total carotenoids, lutein + zeaxanthin, or β-cryptoxanthin in plasma.

		Total carotenoids (n=1006)			Lutein (n=1006)			β-cryptoxanthin (n=1006)		
**Immune cell counts**[2]	Adjustment model	Effect size (95% CI)[1]	Adjusted r^2	*p*-value	Effect size (95% CI)[1]	Adjusted r^2	*p*-value	Effect size (95% CI)[1]	Adjusted r^2	*p*-value
Leukocytes, $\times 10^6$cells/L	Model 0	-7.24 (-8.95; -5.51)	0.058	<0.001	-4.44 (-6.23; -2.60)	0.020	<0.001	-4.42 (-6.21; -2.60)	0.021	<0.001
	Model 1	-6.90 (-8.69; -5.08)	0.063	<0.001	-4.08 (-5.90; -2.24)	0.032	<0.001	-3.94 (-5.78; -2.06)	0.030	<0.001
	Model 2	-4.45 (-6.46, -2.33)	0.106	<0.001	-1.81 (-3.77, 0.20)	0.093	0.077	-1.65 (-3.59, 0.34)	0.093	0.104
Lymphocytes, $\times 10^6$cells/L	Model 0	-2.65 (-4.74; -0.51)	0.005	0.015	-0.90 (-3.04; 1.29)	0.000	0.416	-0.84 (-2.98; 1.33)	0.000	0.444
	Model 1	-3.30 (-5.47; -1.08)	0.008	0.004	-1.12 (-3.28; 1.09)	0.000	0.317	-1.22 (-3.41; 1.01)	0.000	0.280
	Model 2	-1.55 (-4.02; 0.98)	0.043	0.228	0.11 (-2.23, 2.50)	0.042	0.929	0.71 (-1.61; 3.09)	0.042	0.552
Granulocytes, $\times 10^6$cells/L	Model 0	-9.55 (-11.72; -7.32)	0.060	<0.001	-6.28 (-8.58; -3.92)	0.025	<0.001	-6.33 (-8.62; -3.99)	0.025	<0.001
	Model 1	-8.96 (-11.24; -6.62)	0.066	<0.001	-5.76 (-8.08; -3.38)	0.038	<0.001	-5.61 (-7.97; -3.20)	0.036	<0.001
	Model 2	-6.06 (-8.69; -3.36)	0.096	<0.001	-2.89 (-5.44; -0.28)	0.083	0.030	-3.06 (-5.57; -0.48)	0.084	0.020
Monocytes, $\times 10^6$cells/L	Model 0	-8.02 (-10.17; -5.82)	0.045	<0.001	-4.69 (-6.96; -2.37)	0.014	<0.001	-4.99 (-7.24; -2.69)	0.016	<0.001
	Model 1	-5.69 (-7.94; -3.38)	0.088	<0.001	-3.39 (-5.64; -1.09)	0.075	0.004	-2.96 (-5.25; -0.62)	0.073	0.014
	Model 2	-3.12 (-5.69; -0.49)	0.114	0.021	-1.11 (-3.56; 1.39)	0.110	0.381	-0.70 (-3.13; 1.79)	0.110	0.576

Model 0 – No adjustment.

Model 1 – Adjusted for age and gender.

Model 2 – Adjusted for age, gender, antihypertensive medication, smoking status, abdominal obesity, moderate and vigorous physical activity, self-reported diabetes, plasma levels of total cholesterol, and estimated dietary intake of polyunsaturated fatty acids.

[1] The effect size represents the percentage change in the immune cell counts with each increasing tertile of carotenoids.

[2] All markers were log-transformed $\log_{10}$ before regression analysis.

CI, confidence interval.

Table S7. Linear regression models between immune cell counts and tertiles of lycopene, α-carotene, or β-carotene in plasma.

Immune cell counts[2]	Adjustment model	Lycopene (n=1006) Effect size (95% CI)[1]	Adjusted r^2	*p*-value	α-carotene (n=1006) Effect size (95% CI)[1]	Adjusted r^2	*p*-value	β-carotene (n=1006) Effect size (95% CI)[1]	Adjusted r^2	*p*-value
Leukocytes, $\times 10^6$cells/L	Model 0	-5.15 (-6.92; -3.35)	0.028	<0.001	-5.99 (-7.74; -4.22)	0.039	<0.001	-7.32 (-9.02; -5.58)	0.060	<0.001
	Model 1	-4.80 (-6.58; -2.98)	0.039	<0.001	-5.52 (-7.33; -3.68)	0.046	<0.001	-7.11 (-8.93; -5.26)	0.064	<0.001
	Model 2	-3.20 (-4.94; -1.08)	0.099	0.003	-2.71 (-4.68; -0.70)	0.096	0.009	-4.45 (-6.50; -2.36)	0.106	<0.001
Lymphocytes, $\times 10^6$cells/L	Model 0	-2.19 (-4.29; -0.04)	0.003	0.046	-2.37 (-4.47; -0.23)	0.004	0.030	-3.30 (-5.37; -1.17)	0.008	0.003
	Model 1	-2.19 (-4.31; -0.03)	0.003	0.047	-2.87 (-5.03; -0.67)	0.006	0.011	-4.24 (-6.42; -2.01)	0.013	<0.001
	Model 2	-1.52 (-3.79; 0.81)	0.043	0.200	-0.70 (-3.05; 1.71)	0.042	0.566	-1.92 (-4.39; 0.61)	0.044	0.135
Granulocytes, $\times 10^6$cells/L	Model 0	-6.52 (-8.81; -4.18)	0.027	<0.001	-7.86 (-10.10; -5.57)	0.040	<0.001	-9.40 (-11.58; -7.17)	0.058	<0.001
	Model 1	-6.04 (-8.34; -3.69)	0.040	<0.001	-7.12 (-9.44; -4.74)	0.048	<0.001	-8.92 (-11.25; -6.54)	0.064	<0.001
	Model 2	-3.71 (-6.20; -1.15)	0.086	0.005	-3.88 (-6.44; -1.26)	0.086	0.004	-6.04 (-8.68; -3.32)	0.095	<0.001
Monocytes, $\times 10^6$cells/L	Model 0	-5.25 (-7.49; -2.95)	0.018	<0.001	-7.60 (-9.76; -5.38)	0.040	<0.001	-7.89 (-10.05; -5.69)	0.043	<0.001
	Model 1	-4.50 (-6.70; -2.24)	0.081	0.001	-5.50 (-7.73; -3.22)	0.087	<0.001	-5.28 (-7.59; -2.92)	0.084	<0.001
	Model 2	-2.50 (-4.88; -0.06)	0.113	0.045	-2.98 (-5.41; -0.49)	0.114	0.019	-2.81 (-5.39; -0.15)	0.113	0.039

Model 0 – No adjustment.

Model 1 – Adjusted for age and gender.

Model 2 – Adjusted for age, gender, antihypertensive medication, smoking status, abdominal obesity, moderate and vigorous physical activity, self-reported diabetes, plasma levels of total cholesterol, and estimated dietary intake of polyunsaturated fatty acids.

[1] The effect size represents the percentage change in the immune cell counts with each increasing tertile of carotenoids.

[2] All markers were log-transformed $\log_{10}$ before regression analysis.

CI, confidence interval.

Table S8. Linear regression models between levels of inflammatory markers and tertiles of total carotenoids, lutein, or β-cryptoxanthin in plasma.

		Total carotenoids (n=1006)			Lutein (n=1006)			β-cryptoxanthin (n=1006)		
**Inflammatory markers**[2]	Adjustment model	Effect size (95% CI)[1]	Adjusted r^2	*p*-value	Effect size (95% CI)[1]	Adjusted r^2	*p*-value	Effect size (95% CI)[1]	Adjusted r^2	*p*-value
CRP, mg/L	Model 0	-23.84 (-29.00; -18.31)	0.054	<0.001	-17.11 (-22.83; -10.97)	0.025	<0.001	-14.32 (-20.23; -7.98)	0.017	<0.001
	Model 1	-25.62 (-30.85; -20.00)	0.068	<0.001	-17.49 (-23.21; -11.35)	0.036	<0.001	-15.18 (-21.15; -8.76)	0.028	<0.001
	Model 2	-20.57 (-26.77; -13.85)	0.104	<0.001	-12.69 (-19.11; -5.76)	0.087	<0.001	-10.21 (-16.75; -3.15)	0.083	0.005
IL-6, pg/mL	Model 0	-3.87 (-8.21; 0.67)	0.002	0.094	-2.50 (-6.92; 2.14)	0.000	0.285	-0.14 (-4.64; 4.58)	-0.001	0.952
	Model 1	-4.45 (-8.94; 0.27)	0.012	0.064	-2.77 (-7.20; 1.88)	0.010	0.238	-0.48 (-5.08; 4.34)	0.009	0.841
	Model 2	-2.49 (-7.67; 2.98)	0.014	0.365	-1.15 (-6.05; 4.00)	0.014	0.654	1.16 (-3.80; 6.38)	0.014	0.652
IL-18, pg/mL	Model 0	-10.04 (-12.91; -7.08)	0.038	<0.001	-7.61 (-10.59; -4.52)	0.021	<0.001	-8.50 (-11.43; -5.48)	0.027	<0.001
	Model 1	-6.54 (-9.57; -3.40)	0.091	<0.001	-5.74 (-8.71; -2.68)	0.088	<0.001	-5.60 (-8.61; -2.50)	0.087	<0.001
	Model 2	-6.33 (-9.80; -2.72)	0.091	<0.001	-5.13 (-8.41; -1.74)	0.088	0.003	-5.08 (-8.32; -1.73)	0.088	0.003
MPO, ng/mL	Model 0	-3.47 (-6.72; -0.11)	0.003	0.043	-4.44 (-7.66; -1.10)	0.006	0.010	-2.93 (-6.19; 0.45)	0.002	0.088
	Model 1	-3.34 (-6.74; 0.19)	0.004	0.063	-4.37 (-7.63; -1.00)	0.007	0.011	-2.81 (-6.16; 0.66)	0.003	0.111
	Model 2	-3.40 (-7.26; 0.61)	0.000	0.095	-4.22 (-7.78; -0.52)	0.003	0.026	-2.62 (-6.20; 1.09)	-0.001	0.164
MMP-9, ng/mL	Model 0	-5.33 (-8.80; -1.73)	0.007	0.004	-5.81 (-9.26; -2.22)	0.009	0.002	-4.79 (-8.27; -1.18)	0.006	0.010
	Model 1	-5.68 (-9.29; -1.92)	0.007	0.003	-5.89 (-9.38; -2.27)	0.008	0.002	-4.99 (-8.55; -1.28)	0.005	0.009
	Model 2	-4.79 (-8.90; -0.49)	0.006	0.030	-4.73 (-8.57; -0.73)	0.007	0.021	-4.14 (-7.95; -0.17)	0.006	0.041

Model 0 – No adjustment.
Model 1 – Adjusted for age and gender.
Model 2 – Adjusted for age, gender, antihypertensive medication, smoking status, abdominal obesity, moderate and vigorous physical activity, self-reported diabetes, plasma levels of total cholesterol, and estimated dietary intake of polyunsaturated fatty acids.
[1] The effect size represents the percentage change in the inflammatory markers with each increasing tertile of carotenoids.
[2] All markers were log-transformed log_{10} before regression analysis.
CI, confidence interval; CRP, C-reactive protein; IL, Interleukin; MPO, Myeloperoxidase; MMP-9, Matrix metalloproteinase-9.

Table S9. Linear regression models between levels of inflammatory markers and tertiles of lycopene, α-carotene, or β-carotene in plasma.

		**Lycopene (n=1006)**			**α-carotene (n=1006)**			**β-carotene (n=1006)**		
**Inflammatory markers**[2]	Adjustment model	Effect size (95% CI)[1]	Adjusted r^2	*p*-value	Effect size (95% CI)[1]	Adjusted r^2	*p*-value	Effect size (95% CI)[1]	Adjusted r^2	*p*-value
CRP, mg/L	Model 0	-16.54 (-22.28; -10.37)	0.023	<0.001	-19.45 (-24.96; -13.54)	0.034	<0.001	-24.69 (-29.78; -19.22)	0.058	<0.001
	Model 1	-15.60 (-21.43; -9.33)	0.030	<0.001	-20.44 (-26.03; -14.42)	0.045	<0.001	-27.57 (-32.73; -22.02)	0.077	<0.001
	Model 2	-11.26 (-17.72; -4.30)	0.085	0.002	-13.79 (-20.20; -6.85)	0.082	<0.001	-21.48 (-27.62; -14.81)	0.107	<0.001
IL-6, pg/mL	Model 0	-2.05 (-6.48; 2.58)	0.000	0.379	-4.64 (-8.94; -0.14)	0.003	0.044	-2.75 (-7.14; 1.85)	0.000	0.237
	Model 1	-1.37 (-5.83; 3.30)	0.009	0.559	-5.11 (-9.52; -0.49)	0.014	0.031	-3.61 (-8.22; 1.24)	0.011	0.142
	Model 2	-0.51 (-5.38; 4.61)	0.014	0.841	-3.16 (-8.02; 1.97)	0.015	0.223	-1.17 (-6.46; 4.42)	0.014	0.674
IL-18, pg/mL	Model 0	-3.98 (-7.10; -0.76)	0.005	0.016	-9.92 (-12.79; -6.95)	0.037	<0.001	-10.47 (-13.32; -7.52)	0.042	<0.001
	Model 1	-3.30 (-6.34; -0.16)	0.080	0.039	-6.84 (-9.83; -3.76)	0.092	<0.001	-6.47 (-9.56; -3.27)	0.090	<0.001
	Model 2	-2.35 (-5.70; 1.11)	0.082	0.181	-6.41 (-9.69; -3.02)	0.092	<0.001	-5.99 (-9.49; -2.35)	0.090	0.002
MPO, ng/mL	Model 0	-2.25 (-5.54; 1.16)	0.001	0.193	-0.80 (-4.13; 2.65)	-0.001	0.644	-3.33 (-6.58; 0.03)	0.003	0.052
	Model 1	-1.89 (-5.22; 1.55)	0.002	0.277	-0.43 (-3.89; 3.15)	0.001	0.809	-3.30 (-6.76; 0.28)	0.004	0.071
	Model 2	-1.87 (-5.48; 1.87)	-0.002	0.322	0.52 (-3.27; 4.46)	-0.003	0.791	-3.39 (-7.26; 0.64)	0.000	0.098
MMP-9, ng/mL	Model 0	-3.50 (-7.04; 0.18)	0.003	0.062	-2.39 (-5.96; 1.32)	0.001	0.204	-5.20 (-8.67; -1.60)	0.007	0.005
	Model 1	-3.30 (-6.87; 0.42)	0.001	0.081	-2.41 (-6.10; 1.43)	0.000	0.216	-5.76 (-9.42; -1.95)	0.007	0.003
	Model 2	-2.10 (-6.00; 1.96)	0.003	0.306	-0.89 (-4.94; 3.34)	0.002	0.675	-5.31 (-9.41; -1.02)	0.008	0.016

Model 0 – No adjustment.

Model 1 – Adjusted for age and gender.

Model 2 – Adjusted for age, gender, antihypertensive medication, smoking status, abdominal obesity, moderate and vigorous physical activity, self-reported diabetes, plasma levels of total cholesterol, and estimated dietary intake of polyunsaturated fatty acids.

[1] The effect size represents the percentage change in the inflammatory markers with each increasing tertile of carotenoids.

[2] All markers were log-transformed $\log_{10}$ before regression analysis.

CI, confidence interval; CRP, C-reactive protein; IL, Interleukin; MPO, Myeloperoxidase; MMP-9, Matrix metalloproteinase-9.

Table S10. Mediation analysis of the association between total carotenoids in plasma as exposure variable (X), different inflammatory markers as outcome variables (Y), and different types of immune cell counts as mediator variables (M).

			X→M<br>Plasma carotenoid → Cell counts		M→Y<br>Cell counts → Inflammatory marker		X→Y (Total effect)<br>Plasma carotenoid → Inflammatory marker		X→Y (Mediated by M)<br>Plasma carotenoid → Inflammatory marker (mediated by cell counts)		Mediated effect		
**Plasma carotenoid (X)**	**Inflammatory marker (Y)**	**Cell type (M)**	β	*p*-value	β	*p*-value	β	*p*-value	β	*p*-value	β	*p*-value	Proportion of total effect (%)
Total carotenoids	CRP	Leukocyte count	-0.081	<0.001	0.805	<0.001	-0.427	<0.001	-0.362	<0.001	-0.065	<0.001#	15.3
Total carotenoids	CRP	Lymphocyte count	-0.043	0.083	0.142	0.166	-0.427	<0.001	NQ	NQ	NQ	NQ	NQ
Total carotenoids	CRP	Granulocyte count	-0.103	<0.001	0.663	<0.001	-0.427	<0.001	-0.359	<0.001	-0.068	<0.001#	16.0
Total carotenoids	CRP	Monocyte count	-0.062	0.019	0.369	<0.001	-0.427	<0.001	-0.405	<0.001	-0.023	0.050	5.3
Total carotenoids	IL-18	Leukocyte count	-0.080	<0.001	0.149	0.008	-0.152	<0.001	-0.140	<0.001	-0.012	0.033	7.8
Total carotenoids	IL-18	Granulocyte count	-0.102	<0.001	0.118	0.005	-0.152	<0.001	-0.140	<0.001	-0.012	0.030	7.9
Total carotenoids	IL-18	Monocyte count	-0.061	0.021	0.151	<0.001	-0.152	<0.001	-0.143	<0.001	-0.009	0.063	6.1
Total carotenoids	MMP-9	Granulocyte count	-0.108	<0.001	0.624	<0.001	-0.095	0.028	-0.028	0.486	-0.067	<0.001#	70.8
Total carotenoids	MMP-9	Monocyte count	-0.063	0.018	0.484	<0.001	-0.095	0.028	-0.065	0.118	-0.030	0.022	31.9

All regression analyses were conducted on log-transformed variables and represented by the log-transformed β-coefficient.
Significant mediated effect $p<0.017$.
β, β-coefficient from regression analysis indicating effect size; X, exposure variable; Y, outcome variable; M, mediator variable; CRP, C-reactive protein; MMP-9, Matrix metalloproteinase-9; IL-18, Interleukin-18; NQ, Not qualified.

Table S11. Mediation analysis of the association between individual plasma carotenoids as exposure variable (X), different inflammatory markers as outcome variables (Y), and granulocyte count as mediator variables (M).

			**X→M**		**M→Y**		**X→Y (Total effect)**		**X→Y (Mediated by M)**		**Mediated effect**		
			Plasma carotenoid → Cell counts		Cell counts → Inflammatory marker		Plasma carotenoid → Inflammatory marker		Plasma carotenoid → Inflammatory marker (mediated by cell counts)				
**Plasma carotenoid (X)**	**Inflammatory marker (Y)**	**Cell type (M)**	β	*p*-value	β	*p*-value	β	*p*-value	β	*p*-value	β	*p*-value	Proportion of total effect (%)
Lutein	CRP	Granulocyte count	-0.070	0.007	0.687	<0.001	-0.280	<0.001	-0.232	0.002	-0.048	0.011[#]	17.2
β-cryptoxanthin	CRP	Granulocyte count	-0.039	0.014	0.695	<0.001	-0.125	0.006	-0.098	0.026	-0.027	0.020	21.5
Lycopene	CRP	Granulocyte count	-0.055	0.032	0.696	<0.001	-0.213	0.004	-0.175	0.015	-0.038	0.041	18.0
α-carotene	CRP	Granulocyte count	-0.043	0.004	0.684	<0.001	-0.169	<0.001	-0.139	0.001	-0.030	0.008[#]	17.5
β-carotene	CRP	Granulocyte count	-0.059	<0.001	0.661	<0.001	-0.279	<0.001	-0.240	<0.001	-0.039	0.002[#]	13.9
Lutein	MPO	Granulocyte count	-0.080	0.002	0.508	<0.001	-0.088	0.019	-0.047	0.183	-0.041	0.003[#]	46.6
β-cryptoxanthin	MPO	Granulocyte count	-0.042	0.007	0.510	<0.001	-0.043	0.057	NQ	NQ	NQ	NQ	NQ
Lycopene	MPO	Granulocyte count	-0.058	0.025	0.514	<0.001	-0.035	0.337	NQ	NQ	NQ	NQ	NQ
α-carotene	MPO	Granulocyte count	-0.045	0.003	0.519	<0.001	-0.004	0.872	NQ	NQ	NQ	NQ	NQ
β-carotene	MPO	Granulocyte count	-0.062	<0.001	0.513	<0.001	-0.036	0.138	NQ	NQ	NQ	NQ	NQ
Lutein	MMP-9	Granulocyte count	-0.074	0.005	0.621	<0.001	-0.112	0.006	-0.066	0.077	-0.046	0.006[#]	40.9
β-cryptoxanthin	MMP-9	Granulocyte count	-0.041	0.009	0.622	<0.001	-0.063	0.010	-0.037	0.098	-0.026	0.011[#]	40.9
Lycopene	MMP-9	Granulocyte count	-0.058	0.025	0.627	<0.001	-0.045	0.258	NQ	NQ	NQ	NQ	NQ
α-carotene	MMP-9	Granulocyte count	-0.045	0.004	0.632	<0.001	-0.009	0.696	NQ	NQ	NQ	NQ	NQ
β-carotene	MMP-9	Granulocyte count	-0.061	<0.001	0.626	<0.001	-0.047	0.072	NQ	NQ	NQ	NQ	NQ

All regression analyses were conducted on log-transformed variables and represented by the log-transformed β-coefficient.

Significant mediated effect $p<0.017$.

β, β-coefficient from regression analysis indicating effect size; X, exposure variable; Y, outcome variable; M, mediator variable; CRP, C-reactive protein; MPO, Myeloperoxidase; MMP-9, Matrix metalloproteinase-9; NQ, Not qualified.
